# Radical framing effects in the ultimatum game: the impact of explicit culturally transmitted frames on economic decision-making

**DOI:** 10.1098/rsos.170543

**Published:** 2017-12-20

**Authors:** Aaron D. Lightner, Pat Barclay, Edward H. Hagen

**Affiliations:** 1 Department of Anthropology, Washington State University, Pullman, WA, USA; 2 Department of Psychology, University of Guelph, Guelph, Ontario, Canada

**Keywords:** behavioural economics, ultimatum game, cultural evolution, evolutionary psychology

## Abstract

Many studies have documented framing effects in economic games. These studies, however, have tended to use minimal framing cues (e.g. a single sentence labelling the frame), and the frames did not involve unambiguous offer expectations. Results often did not differ substantially from those in the unframed games. Here we test the hypothesis that, in contrast to the modal offer in the unframed ultimatum game (UG) (e.g. 60% to the proposer and 40% to the responder), offers in a UG explicitly framed either as a currency exchange or a windfall will closely conform to expectations for the frame and diverge substantially from the modal offer. Participants recruited from MTurk were randomized into one of two conditions. In the control condition, participants played a standard UG. In the treatment conditions, players were provided a vignette explicitly describing the frame with their roles: some were customers and bankers in a currency exchange, and others were in a windfall scenario. We predicted (i) that modal offers in the currency exchange would involve an asymmetric split where greater than 80% went to customers and less than 20% went to bankers, and (ii) that variation in windfall offers would converge onto a 50–50 split with significantly less variation than the control condition. Our first prediction was confirmed with substantial effect sizes (*d* = 1.09 and *d* = −2.04), whereas we found no evidence for our second prediction. The first result provides further evidence that it is difficult to draw firm conclusions about economic decision-making from decontextualized games.

## 1. Introduction

For decades, economists have made game theoretic predictions about cooperative decision-making behaviour among humans based on traditional assumptions of self-interested utility maximization, where utility is defined as individual benefit such as monetary profit or material incentives [1]. Empirical results from experimental economic games, such as the ultimatum game (UG), seem to challenge these assumptions [2–4] but have been criticized for failing to take cultural context into consideration [5,6]. In the UG, typical experimental outcomes deviate from a subgame perfect equilibrium and rarely conform to Nash equilibria without several iterations of game play [7,8]. Substantial deviations from a subgame perfect equilibrium occur across cultures, regions, economic systems and subsistence strategies, with the magnitude of the deviations varying among groups. Even within groups, however, there is substantial individual variation in both offers and acceptance thresholds [9,10].

There are at least two mutually compatible explanations for individual variation in the UG within groups. First, criticisms of the one-shot UG methodology have noted that individuals only converge onto Nash equilibria insofar as they are able to learn the game through several rounds of play [11]. This means that high variance in UG outcomes might simply reflect this learning curve [12,13], but carries an important implication: that, in virtually all games and real-world settings, individuals converge to equilibria through learning [14]. Second, typical UG experiments provide each player with the rules, but no other context for game play. In the absence of almost any contextual cues, individuals might construct different internal models of the UG with different criteria for offers and acceptance thresholds. If so, the high levels of individual variation in standard UG outcomes is largely a result of different people essentially playing different games, even though the rules are objectively identical [5].

Human rationality is subject to constraints. Many theorists argue that because finding optimal solutions to many decision problems by rational thought is not computationally tractable, agents instead execute strategies that are optimized either by natural selection or by individual or social learning [15,16]. On this view, the brain often acts as a strategy executor rather than as a strategy optimizer, and the strategies are relevant to the environments for which they were optimized (genetically or ontogenetically). This means, however, that to execute the best strategy, the brain must first accurately identify the environment, a problem often referred to as the ‘frame problem’ [17]. Humans appear to solve this problem by using environmental cues to cognitively access a broader semantic network of scripts (acquired via natural selection or individual or social learning), forming beliefs and expectations appropriate to a given context [18–20], which is often referred to as ‘framing’ the situation.

Contrary to traditional views of rational decision-making, the cognitive challenge in standard UG games might not be the optimization of a strategy, but the identification of the correct frame (e.g. [5]). This has also been referred to as a ‘logic of appropriateness’, which requires a person to identify the correct social context (i.e. internally ‘asking oneself’ what they are meant to do in a given social situation), before processing the appropriate heuristic(s) and executing associated behavioural strategies [21]. This challenge is especially acute in most UG experiments because study participants are only given the rules and no other information. In such informationally sparse conditions, the context of the game is inherently ambiguous. Is the game a competition, for instance, or a cooperative exchange? Does it involve friends or enemies? In the face of such ambiguity, participants might choose different frames based on differences in their ‘state’, e.g. differences in their personal experiences, personal circumstances, or socially learned concepts. The choice of frame might even have a substantial stochastic component. If, as seems likely, different individuals frame the UG differently, they could be executing different strategies that involve substantially different offers and acceptance thresholds.

This hypothesis can be tested by explicitly framing the UG with rich descriptions of a familiar economic context, which would allow study participants to avoid the challenge of framing the very abstract rules of a one-shot UG, and instead focus on picking the optimal strategy for that economic context, e.g. based on prior experiences. This should result in acceptable offers which reflect the expectations relevant to the given explicit frame, with less individual variation in game play [5].

Several studies have explored the impact of environmental cues on play in economic games. Haley & Fessler [22], for example, found that the presence or absence of eye-spots altered offers in the Dictator game (DG), and Charness & Gneezy [23] further showed that cues decreasing social distance between players, such as providing their names, did the same in the UG. Liberman *et al*. [24] demonstrated that cooperation in a public goods game (PGG) with dichotomous choices (i.e. cooperate or defect) among US students was more than twice as likely when it was called a ‘community game’ than when it was called a ‘Wall Street game’. Similarly, Leliveld *et al*. [25] found that framing the proposers' offers as ‘giving’ versus ‘taking’ also altered offers in the UG. While such cues narrow the choice of frames, they do not necessarily indicate a single ‘best’ frame because there are typically only one or two cues, and they are consistent with multiple frames. For example, eye-spots do not uniquely determine any one particular frame, ‘giving’ is consistent with both gifting and with trade, and ‘community game’ is ambiguous.

Previous studies have noted the importance of culturally relevant frames. For example, in a study involving the PGG among the Kenyan Orma people, Henrich *et al.* [9] observed that the participants identified the PGG as a ‘harambee’ game. This was in reference to a group welfare-oriented institution structured similarly to the PGG. Indeed, participants in this study population played with high rates of cooperation. Other studies have investigated the impact of intentionally providing participants with a single, explicit frame (table 1). While this has potentially eliminated some ambiguity, these studies typically only provide (at most) a limited set of cues to individuals regarding a culturally specific institution, of which participants have an intimate knowledge (e.g. because it is culturally valued, relevant to subsistence, or provided to participants before game play) [28,31]. Cronk [6], for example, conducted a trust game among the Maasai of Kenya, who have an economic institution called ‘osotua’. Osotua is deeply important to the Maasai, and pervades the fabric of their society with indefinite, need-based gift giving relationships. In the instructions, Cronk [6] included a single sentence that identified the institution: ‘This is an osotua game’. In another example, Gerkey [30] conducted a PGG among salmon fishers and reindeer herders in Siberia, who have collective institutions for resource sharing called ‘sovkhoz’ and ‘obshchina’. The framed versions began with the sentence ‘This game is called the sovkhoz/obshchina game’, and participants could contribute to the ‘sovkhoz fund’ or ‘obshchina fund’. Although these explicit frames did have modest effects on mean offers (table 1—see also [27,29]), the limited nature of the cues might have still left considerable scope for different interpretations by the participants. In some studies, the standard deviation of offers increased in the framed versus unframed conditions.

**Table 1.** Previous economic game studies investigating framing effects.

participants (country)	frame	mean (s.d.) portion transferred	type of game	citation
students (Germany)	standard	0.363 (0.133)	ultimatum game	Guth *et al.* [26]
students (Netherlands)	standard	0.602 (0.098)	ultimatum game	Leliveld *et al.* [25]
students (Netherlands)	‘take’, ‘split’, ‘give’ (offers, respectively)	0.641 (0.123)	ultimatum game	Leliveld *et al.* [25]
		0.596 (0.119)		
		0.490 (0.194)		
students (US)	‘dividing’, ‘claiming’ (minimally acceptable offers, respectively)	0.294 (0.214)	ultimatum game	Larrick & Blount [27]
		0.279 (0.204)		
Maasai (Kenya)	standard	0.353 (0.191)	trust game	Cronk [6]
Maasai (Kenya)	osotua	0.282 (0.161)	trust game	Cronk [6]
Maasai (Kenya) and students (US)	standard	0.564 (0.287)	trust game	Cronk & Wasielewski [28]
Maasai (Kenya) and students (US)	osotua	0.446 (0.279)	trust game	Cronk & Wasielewski [28]
Amazon Mechanical Turk (US)	‘teamwork game’, ‘paying taxes game’	0.569 (0.264)	public goods game	Eriksson & Strimling [29]
		0.407 (0.262)		
Kamchatka (Russia)	standard	0.974 (0.105)	public goods game	Gerkey [30]
Kamchatka (Russia)	‘sovkhoz’ plus ‘obshchina’	0.859 (0.246)	public goods game	Gerkey [30]
students (Netherlands)	‘partners’ and ‘strangers’	0.190 (0.305)	public goods game	Keser & van Winden [31]
		0.453 (0.395)		

Interestingly, large-scale market economies are often composed of many similar gift-based traditions, such as birthday and Christmas gifting, along with impersonal, price-driven institutions, such as the stock market. A cultural frame relating to the latter is less relevant to other-regarding considerations (e.g. reciprocity, generosity) and more relevant to the price or percentage on which society has converged for efficiency in that given exchange scenario [32,33]. Some socially learned frames of this kind might have ‘fair’ offer values close to zero (e.g. a currency exchange transaction fee), but in a windfall scenario between friends, an offer deviating from a 50% split might be considered unfair and evoke negative emotions. Furthermore, if a population-level consensus about a ‘fair’ offer percentage in a given frame were common knowledge to participants, then this offer value would ideally emerge with low individual variation. However, the social pressure to become familiar with certain formal economic institutions, such as currency exchange for international travel, is low in contrast to subsistence collectives or culturally valued concepts like osotua, or even birthday and Christmas gift-giving traditions. This suggests that fair offers in some market-related frames might be associated with a wide spectrum of familiarity. In other words, people learn these frames to varying degrees. The consequence of this would then be that the emergence of low individual variation would also be moderated by familiarity with the frame.

### 1.1. Study aims and hypotheses

In a standard one-shot unframed UG, mean offers are typically 40–50%, and most of these are accepted, but smaller offers are rarely accepted [2,8]. This study will test the hypotheses that: (i) mean offers and acceptance thresholds in a one-shot UG that is explicitly framed as a transaction within a familiar economic institution will closely conform to societal norms for that institution, and (ii) variation of offers will significantly decrease when a standard UG is explicitly framed as a windfall gift scenario. Specifically, if normative offers are less than 10%, e.g. bank fees, then average offers will be less than 10%, and most of these will be accepted. Conversely, if normative offers are greater than 90%, then average offers will be greater than 90% and offers between 50–90% will be at higher risk of rejection. This would constitute both a substantial and bidirectional deviation from patterns seen in most one-shot UG studies. Furthermore, if the one-shot UG is commonly, but not always, viewed as a windfall sharing scenario, as previous literature suggests, then we expect that providing a more detailed description of such a scenario might yield a narrower distribution of offers. This would be a consequence of reduced ambiguity, and variation would largely reflect differences in sharing behaviour alone, in contrast to sharing behaviour plus potential confusion about the UG. In summary, by framing the UG as a familiar economic institution, participants will already have learned the relevant ‘fair’ offers in real life, and so we predict their offers will conform to these norms without any learning in the game itself.

For this study, we will focus on patterns of play in UGs explicitly framed as a currency exchange, an economic institution that is common in the US and other Westernized societies. The currency market is a multi-level exchange market in which the interbank market, made up of large banks and financial institutions, constitute the top tier and trade currencies in high volumes at floating exchange rates, determined by extremely narrow spreads (i.e. markups, or differences between bids and ask prices). When currency exchange transactions reach an individual customer, transaction fees are common in the form of deviations from the ‘true’ foreign exchange rate offered to the interbank market, explicitly stated transaction fees, or often both [34,35]. Banks typically profit from spread differences alone in liquid currency exchange transactions, but explicitly stated transaction fees are often applied to credit card transactions involving currency exchange [36].

Wholesale dual currency investments are often as low as 0.10–0.15% after being assessed by banks, and competitive bank fees applied to day-to-day transactions involving currency exchange are often roughly 1.5–2% (e.g. Citibank and Wells Fargo websites). Prevalent credit card services in the US typically assume an estimated range of bank fees to be 0–5%. While banks are a widespread source of currency exchange to most individuals, other services commonly encountered include private companies (e.g. Travelex) with airport locations profiting mostly from their convenience of use, offering $7.95 service fees or 2% (whichever sum is higher) on sums under $500. This can deviate substantially from common bank fees of 1–5% in some cases (e.g. a $10 exchange incurs a 79.5% transaction fee), but because currency exchange is typically carried out in large quantities, these rates are probably rarely encountered and commonly recognized as unfair. In fact, the 2% lower limit in this case seems to be an indicator of the common customers' demand to push currency exchange transaction fees down to near zero.

Our hypotheses will be tested by comparing patterns of game play in a standard unframed one-shot UG (the control condition), to patterns in a one-shot UG explicitly framed as either a windfall gift scenario or a detailed currency conversion scenario (the treatment conditions). In the windfall, allocations will be referred to as a ‘bonus gift’ to be fairly split between the proposer and responder. In the currency exchange, allocations will be referred to as ‘bank fees’ between the proposer and responder. In one currency exchange condition, the proposer will be referred to as the ‘banker’, and the responder will be referred to as the ‘customer’. In another currency exchange condition, the roles will be switched. Framing the UG will not modify the rules or abstract structure of the game, but could yield large effect sizes in the currency exchange conditions, and a relatively narrow distribution of acceptable offers in the windfall condition.

In particular, we expect that participants in the windfall treatment will narrowly converge near a 50% split, with a significantly narrower distribution of offers than in the control condition. In both currency exchange treatments, we expect that participants will commonly agree on a banker allocation of less than 20%. This means that proposers labelled as ‘customers’ would offer amounts deemed acceptable by the responder of less than 20%, whereas proposers labelled as ‘bankers’ will commonly offer acceptable transfers of 80% or more. In general, we predict that offers made by proposers will differ substantially from the 40% allocations typically seen in the standard UG in the US and Europe, with chances of acceptance corresponding closely with the range associated with their reported institutional norms. We predict responders in the banker role to frequently accept very low offers, whereas proposers in the banker role will frequently offer hyper-fair offers (i.e. offers higher than 50%) while retaining some risk of rejection from responders in the customer role.

## 2. Methods

All participants were randomly assigned into one of four conditions: control (standard UG), a windfall gift condition, a customer-as-proposer, and a banker-as-proposer condition (the latter two references to proposer labels will henceforth be called ‘customer condition’ and ‘banker condition’, respectively). In the control condition, participants played the standard UG with a pie of $1.00, implemented using a modified version of oTree, an open source platform for online social science experiments [37]. Participants in the proposer role were referred to as ‘player 1’ and participants in the responder role were referred to as ‘player 2’. Player 1 was asked to make an offer to player 2, which s/he could accept or reject.

In the windfall gift condition, the standard UG was framed as a bonus gift to be ‘fairly split’ with another person. In the banker/customer treatment conditions, participants played the UG after reading a brief and detailed vignette framing the UG as a currency exchange scenario. While the instructions outlined the same hypothetical scenario in both treatment conditions, each of these conditions differed in their assignment of roles to player 1 and player 2. In the customer condition, player 1 was instead referred to as the ‘customer’, player 2 was referred to as the ‘banker’, and the offer was called the ‘bank fee’. In the banker condition, player 1 was referred to as the ‘banker’, player 2 was referred to as the ‘customer’, and the offer was the remaining amount transferred after charging a ‘[requested] bank fee’. (See appended instructions and vignettes in the electronic supplementary material.)

After game play ended in all conditions, participants were asked to complete a brief post-experiment questionnaire (see below).

Our study design involved no deception: each condition involved two real players, and each player received payoffs in real money in accordance with the game rules.

### 2.1. Pilot study

To evaluate our study design and validate normative offers in a currency exchange, we piloted our study with a sample of 374 participants on Amazon Mturk (AMT) (full details of the pilot study are reported in the electronic supplementary material). The mean offer in the control condition (standard UG) was 43.5%, s.d. = 20.5% The mean offer in the customer condition was significantly less (mean = 18%, s.d. = 20.5%, *p *< 0.001) than the control, as predicted, and in the banker condition it was significantly higher (mean = 69.0%, s.d. = 32.0%, *p *< 0.001), as predicted. After game play, we asked all participants to state a fair bank fee for currency exchange, and the mean fee was 8.4% (s.d. = 15%).

**Figure 1.**
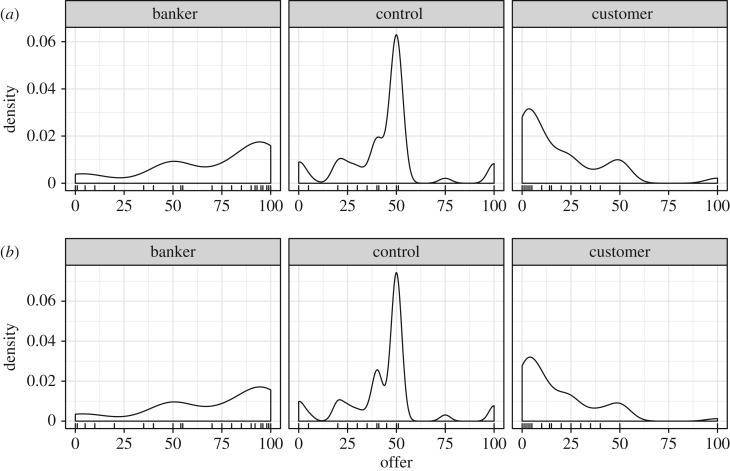
Pilot data. Density plots of all offers by condition, with raw data before exclusion (*a*) and after exclusion (*b*); bandwidth = 9.08, rug indicates offers.

**Figure 2.**
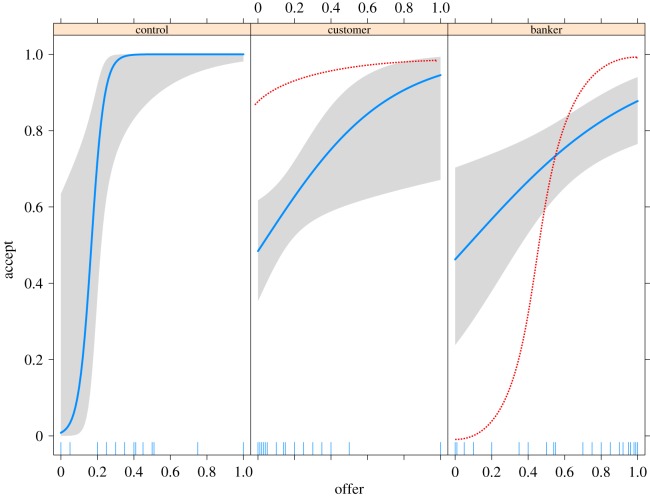
Pilot data. Plot of logistic regression of responders' acceptance probability as a function of offer amount by condition (grey shaded areas indicate 2 s.e., rug indicates offers). Dotted red line overlay indicates our predicted trends for responder acceptance probability in the treatment conditions.

In the pilot study, there were three main deviations from our original predictions. First, the variation of offers did not significantly decrease (customer–control variances Brown–Forsythe *F* = 0.676, *p *= 0.412), contrary to predictions, and the variation (interquartile range (IQR) and s.d.) in the banker condition actually increased relative to the control condition (*F* = 23.2, *p *< 0.001) (figure 1). This deviation from our predicted shifts in variance was robust across levels of familiarity with currency exchange (data are reported in the electronic supplementary material). Second, when participants in the treatment condition were asked what a fair bank fee would be in the game they played, responses were much higher (mean = 31.3%, s.d. = 26.0%, median = 20%), which was in contrast to the reported fair bank fee in real-world scenarios (stated previously). Third, responders in the treatment condition had higher rejection rates than those in the control condition (per cent acceptances in control = 89.6%, customer = 60.2%, banker = 76.8%), contrary to our predictions, though a number of hyper-fair offers in the banker condition were rejected as predicted (figure 2). Taken together, these deviations suggested that we needed to increase the specificity and richness of our vignettes to further reduce ambiguity compared to the vignettes used in our pilot study.

We therefore modified our protocol in two important ways. First, we used more detailed vignettes in the treatment conditions relating to currency exchange. In the pilot study, participants in the treatment condition read the same vignette, after which they were assigned to either the customer or banker role. In our modified study protocol, participants were first assigned to the customer or banker role; participants in the customer role then read a detailed vignette presented from the customer's point of view, and those in the banker role then read a detailed vignette presented from the banker's point of view. Second, we abandoned our prediction that variance would be reduced in the original treatment conditions compared to the control condition. Instead, we included a new ‘windfall’ condition in which we predicted reduced variance relative to our control condition (but we made no predictions about the central tendency of offers in the windfall condition). Like the currency exchange treatment conditions, we presented the windfall instructions as a detailed vignette. Overall, we predicted our modified protocol would increase the treatment effect, relative to results in this pilot study.

### 2.2. Power analysis

Before collecting data, we conducted a power analysis to determine the sample size necessary to detect a difference in central tendency between UG offers in the control and each treatment condition. To do this, we used the empirical pilot data to estimate our anticipated offer distributions in each condition. The effect sizes for these empirical data in both treatment conditions were large (banker condition: Cohen's *d* = −0.95 and Cliff's delta = −0.50; customer condition: Cohen's *d* = 1.24 and Cliff's delta = 0.625—see [38]).

**Figure 3.**
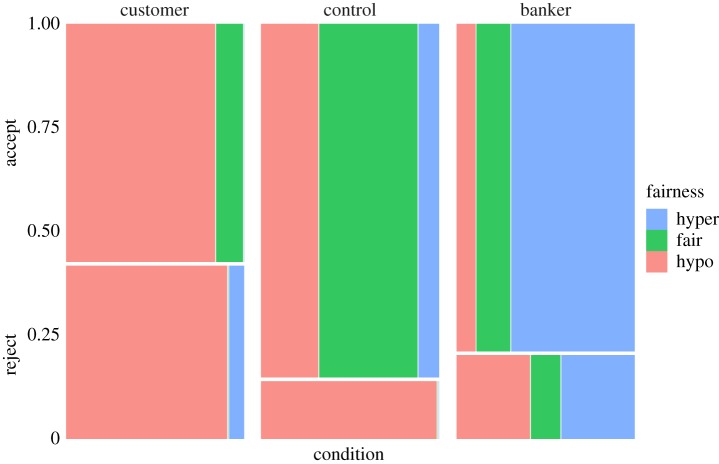
Pilot data. Mosaic plot depicting frequencies of responders' acceptance and rejection of offers under 50% (‘hypo’), at 50% (‘fair’) and over 50% (‘hyper’) by condition.

To estimate a sufficient sample size (i.e. number of UG played in each condition) for detecting an effect on the central tendency of offers between control and treatment conditions, we simulated 10 000 experiments by drawing random samples with replacement from our empirical control and treatment distributions. For each experiment, we computed Mann–Whitney *U* tests of sampled offers, and for each simulation of 10 000 experiments we systematically varied sample sizes (*n* = 3, 4, 5, …, 60). Given our large effect sizes in the empirical data, we determined sufficient sample sizes as low as about *n* = 20–25 games per condition (power > 0.80–0.90, *α* = 0.05) (figure 3).

**Figure 4.**
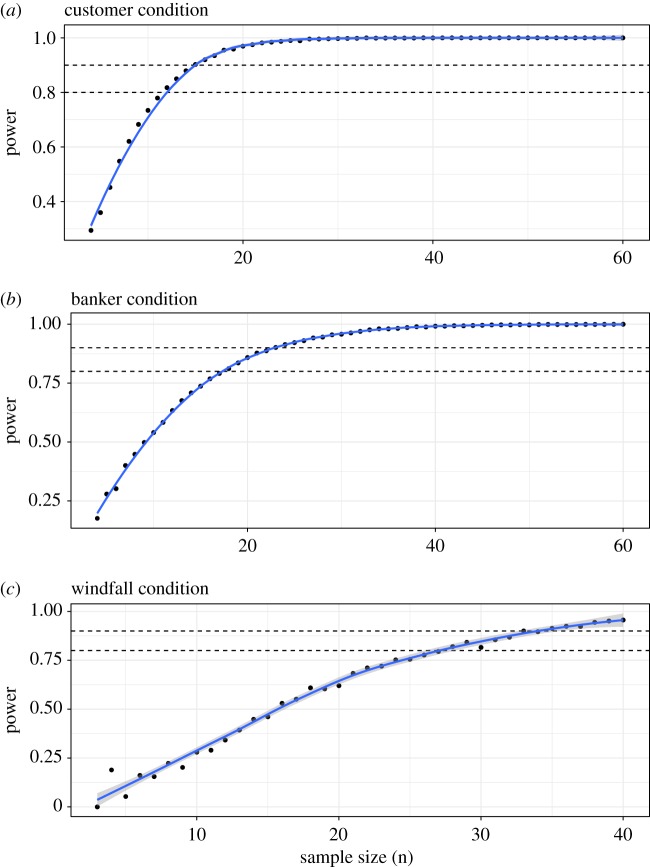
(*a*,*b*) Power as a function of sample size in each currency exchange treatment condition, based on bootstrapped empirical data from pilot study. (*c*) Power as a function of sample size in windfall treatment condition, based on bootstrapped empirical data from pilot study.

For a second power analysis, we determined the appropriate sample size to detect a significant difference in the variance of offers made between the control and windfall (mean = 45, s.d. = 10) conditions using a similar empirical method. We used a Brown–Forsythe Levene-type test for equality of variances [39] in each experiment of a 1000 experiment simulation, systematically varying sample size in each simulation (*n* = 3, 4, 5, …, 40). In this case, we determined sufficient sample sizes around *n* = 30–35 games per condition (power > 0.80–0.90, *α* = 0.05) (figure 4).

The minimum sample size sufficient to detect all of our hypothesized effects, as determined by these power analyses, was *n* = 35 games per condition. This would correspond to at least 70 participants (i.e. 35 proposers and 35 responders, each paired into 70 one-shot UG) in each of the four conditions (*n* = 280 participants in this study). We discuss our (actual) sample size used further in the Sample subsection of this Methods section.

### 2.3. Sample

We recruited 480 participants (60 pairs of participants in each of 4 conditions) on Amazon Mturk to play a one-shot UG with $1 earning stakes in addition to a $0.25 show-up fee. While our study design was informed by the pilot data, we increased our sample sizes in each condition from the values in our power analyses because there is uncertainty in the estimate of effect size in our pilot data (the true effect might be smaller or larger). Similarly, though we included more stringent attention check questions and criteria, this increase in sample size allowed us to account for potential excluded data. Data exclusion criteria were 3 out of 3 incorrect attention check questions, nonsensical responses to the questions about maximum and minimum values (see Variables), and exceptionally low HIT times (less than 90 s).

### 2.4. Variables

For UG outcomes in all conditions, we recorded the amount offered (*s* = [$0.00, 0.01, 0.02, …, 1.00]) by participants in the proposer roles and the acceptance (a = [0, 1], where 0 = reject and 1 = accept) of participants in the responder role. In addition to these UG outcomes, we collected participant demographic information, beliefs and expectations about the game they played, game-related and social preferences, emotional responses to the game, and experiences with currency exchange. These were collected in a post-experiment questionnaire. Age, sex, occupation and nationality were also recorded.

To assess expectations associated with the currency exchange frame, we asked all participants what they considered to be a fair currency exchange fee ‘in real life’, along with potential proxies in case experience was universally low (e.g. fair ATM fees, online service charge fees and broker commission fees). Similarly, we asked all participants what they considered to be a fair split of a bonus gift to assess expectations in the windfall treatment condition.

To address beliefs and expectations about the other participant, participants playing in the proposer role were asked which minimum and maximum offers they thought the other participant expected them to make. They were also asked for their ‘ceiling amount’, i.e. what amount they considered to be their highest acceptable offer (HAO). Similarly, participants in the responder role were asked which minimum and maximum possible offers they expected the proposer was likely to make, along with their minimally acceptable offer (MAO).

To measure individual expectations and preferences during the experiment, all participants were asked what amount they believed was a fair offer in their given context. Specifically, the control and windfall treatment participants were asked what they considered to be a fair ‘offer’ in the experiment, whereas currency exchange treatment participants were asked what they considered to be a fair ‘bank fee’ in the experiment.

To address emotional responses associated with game play outcomes for all participants, we included a question from a previous study [40], asking, ‘how did you feel about the outcome of this game?’ on a 5-point Likert scale (1–5; 1 = very negatively, 5 = very positively). Additionally, we asked participants to evaluate the statement, ‘I thought that the outcome of this game was fair’ on a Likert scale (1–5; 1 = strongly disagree, 5 = strongly agree).

Note that in both of the above sections, the language of each question remained consistent with that of the participant's given frame (e.g. ‘offers’ corresponding to the control condition were still referred to as ‘bank fees’ in treatment conditions, etc.)

To assess experience with currency conversion we asked participants to rank on a Likert scale how much experience they have with converting different types of currency (for example, when travelling—1–5; 1 = no experience, 5 = extensive experience). We also asked participants to rank how much experience they have with travelling internationally (1–5; 1 = no experience, 5 = extensive experience), and what percentage amount they consider a standard and fair bank fee for exchanging currency in real life situations. Lastly, all participants were given a nine-question social value orientation (SVO) survey [41], and their SVO score was used as a measure of social preferences.

While AMT seems to provide more attentive participants than average psychology research subject pools at a university [42], we included an attention check in the instructions by asking participants to click on a picture of a clock tower when they see one. This approach was modelled after attention check questions in [43], and we used incorrect selections on this question as criteria for excluding a participant's data.

### 2.5. Statistical analyses

To analyse the key predictions of this study, we first tested for the predicted central tendency shifts between the control and currency exchange treatment conditions by using parametric and nonparametric tests (ANOVA for all data, *t*-test and Mann–Whitney *U*, respectively for pairwise comparisons). We then tested for changes in individual variation among offers by using both a Fligner–Killeen test [44] and a robust Brown–Forsythe Levene-type test, each of which were to test for equality of variances between control and windfall treatment conditions. More than one test was used for analysing this because they are each suitable for different degrees of deviation from normality. We used generalized linear models to model responder acceptance behaviour as well as proposer behaviour, and to determine any added effect of adding currency exchange experience as a parameter into our model.

Because our pilot data led us to expect a possible heterogeneous distribution of offers, where different subsets of participants play differently, we used GAMLSS in R [45] to characterize mixture models in our treatment conditions. For other variables (i.e. items in the post-experiment questionnaire), we have no strong predictions. We conducted analyses using these variables, mostly surrounding expectations and preferences during game play and social preferences, and we refer to these analyses as strictly exploratory. The purpose of this component of our study was to seek possible directions for future research.

## 3. Results

**Figure 5.**
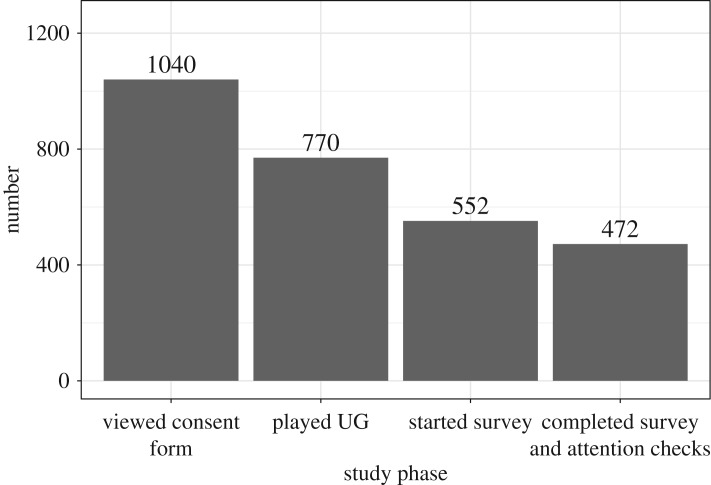
Number of participants during each study phase. Individuals who completed the survey and attention checks (*N* = 472) comprised our ‘included’ sample for analysis.

To achieve 60 pairs of participants per condition after attrition, we recruited 70 pairs of participants per condition. Following our *a priori* criteria, and before analysing the data, participants were excluded if they did not complete the survey, which was determined by a failure to correctly enter a unique validation code at the end of the survey. This was only attainable if a participant correctly answered two attention check questions during the task, prior to reaching the validation code. These three exclusionary criteria were set out to remove from analyses participants less likely to be attentive. We also initially planned to exclude participants who finished the study too quickly (suggesting low attention), as seen in several participants in the pilot study, but because we were able to shorten the final study (by omitting some exploratory items; see below), that criterion did not apply to any participant. In addition, many other participants left the study beforehand, e.g. while the game was loading or after viewing the consent form (figure 5). Attention checks were applied separately to proposers and responders, resulting in slightly different numbers of each (proposers = 228; responders = 244). After exclusions, we had a final sample of *N* = 472, which was slightly smaller than our target of 480. Other than our stated exclusion criteria, we did not make any decisions on sample size based on observing the data. All pilot data (Phase I) and final study data (Phase II) are publicly available [46–48].

Participants in our final sample included 67% from the US, 10% from India, 8.47% from various other regions (e.g. Europe, South America, Africa, Australia, North America non-US, Asia non-India), and 15% unspecified or ambiguous responses. Participant ages ranged from 18 to 75 (mean = 35, s.d. = 11), with 54% male and 46% female. Based on Likert score responses, participants commonly lacked extensive experience with currency exchange (scale of 1–5 with 1 = no experience and 5 = extensive experience: mean = 2.5, s.d. = 1.1) and international travel (mean = 2.5, s.d. = 1.2). See electronic supplementary material, figures S5 and S6.

**Figure 6.**
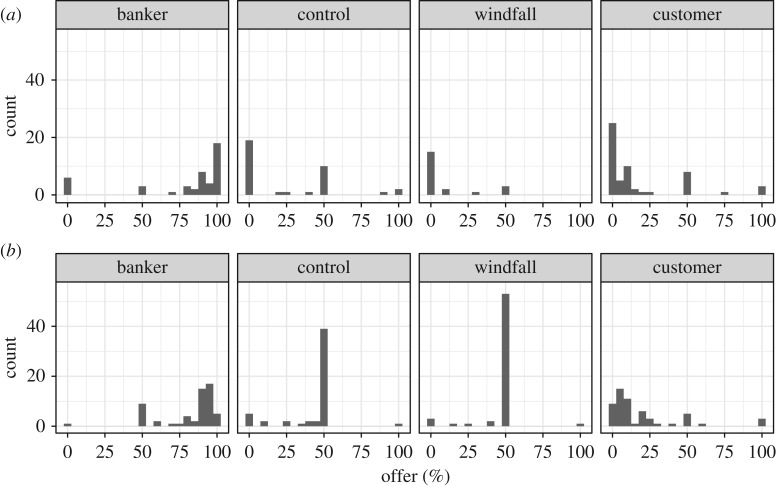
Distribution of proposers' offers by condition. (*a*) Offers of excluded participants. (*b*) Offers of included participants.

We refer to the condition with proposers in the banker role as the ‘banker condition’, and the condition with proposers in the customer role as the ‘customer condition’. In the banker and customer conditions, there did not appear to be a marked difference in the distribution of offers between the excluded participants who played the UG (but did not complete the survey) and the analysed participants who completed the entire study (figure 6). There was a large difference in the distribution of offers from excluded versus included participants in the windfall condition, however, where almost all excluded participants offered 0% but almost all included participants offered 50%, and also in the control condition, where more excluded participants offered 0% than 50% but almost all included participants offered 50%. Other interesting differences included that in the banker condition, a greater fraction of offers from excluded participants were either 0% or 100% compared to offers from included participants, and that in the customer condition a greater fraction of offers from excluded participants were either 0% or 50% compared to included participants (figure 6). Henceforth, unless explicitly noted, all analyses will be of data from included participants only.

**Figure 7.**
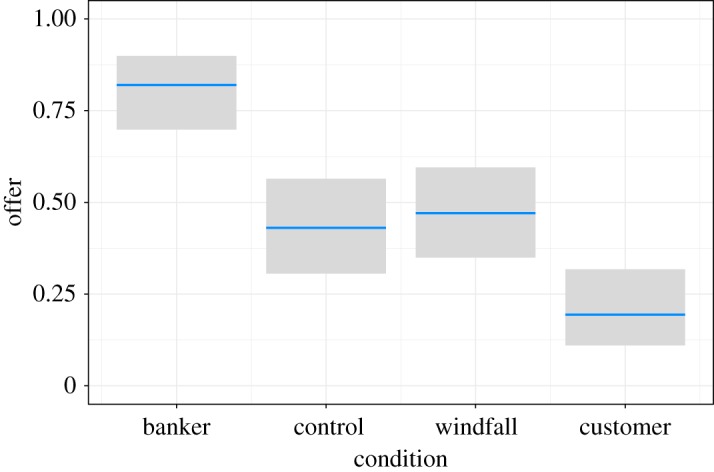
Logistic regression effect plot of the mean offer (as a proportion of the maximum possible offer) by condition. Bars indicate ± 2 s.e. Model coefficients are in table 2. See text for details.

In our experiment, the central tendency differences between treatment and control conditions were significant and conformed to our predictions, with offers in the banker condition (mean = 81.8%, s.d. = 23.1%) significantly higher than the control condition (mean = 42.1%, s.d. = 21.7%, *t* = −13.6, *p *= 1.8 × 10^−31^, Cohen's *d* = −2.04) and offers in the customer condition (mean = 18.7%, s.d. = 24.2%) significantly lower than the control condition (*t* = 7.71, *p *= 3.9 × 10^−13^, Cohen's *d* = 1.09) (table 2 and figure 7).

**Table 2.** Logistic regression model of mean offers by condition. Estimates are log odds. The base condition is the banker condition. Null deviance: 98.2 on 227 degrees of freedom. Residual deviance: 49.4 on 224 degrees of freedom. See figure 7 for an effects plot.

term	estimate	s.e.	statistic	*p*-value
intercept	1.52	0.34	4.40	1.1 × 10^−5^
conditioncontrol	−1.79	0.44	−4.07	4.7 × 10^−5^
conditionwindfall	−1.63	0.43	−3.80	1.4 × 10^−4^
conditioncustomer	−2.94	0.48	−6.09	1.1 × 10^−9^

The data in each condition were not normally distributed (Shapiro–Wilk: banker *W* = 0.738, *p *= 9.4 × 10^−9^; customer *W* = 0.701, *p *= 2.2 × 10^−9^; windfall *W* = 0.444, *p *= 7.7 × 10^−14^; control *W* = 0.62, *p *= 1.4 × 10^−10^). As stipulated in our *a priori* statistical analyses section we therefore also tested for differences in offers using a non-parametric Kruskal–Wallis rank sum test (*χ*^2^ = 124, *p *= 1.2 × 10^−26^). Likewise, pairwise median differences between banker and control conditions (banker median = 90, control median = 50) and customer and control conditions (customer median = 10) were retested to confirm that our predictions were supported for both banker and customer conditions (banker–control Mann–Whitney *U* = 282, *p *= 9.5 × 10^−15^, and Cliff's delta = −0.817; customer–control *U* = 2370, *p *= 1.2 × 10^−7^, and Cliff's delta = 0.565).

While the offer variance in the windfall vignette condition was lower than that of the control condition (windfall mean = 44.8%, s.d. = 16.8%, IQR = 0%; control IQR = 10%), this difference was not significant (*F* = 2.14, *p *= 0.15; *χ*^2^ = 2.69, *p *= 0.1) using either equality of variances test (i.e. Brown–Forsythe Levene-type and Fligner–Killeen tests), contrary to predictions. It is worth noting, however, that the portion of ‘fair splits’ (i.e. 50–50 offers) among proposers was 72.2% with an acceptance rate of 79.6% in the control condition, and 85.2% fair splits with an acceptance rate of 83.6% in the windfall condition.

**Figure 8.**
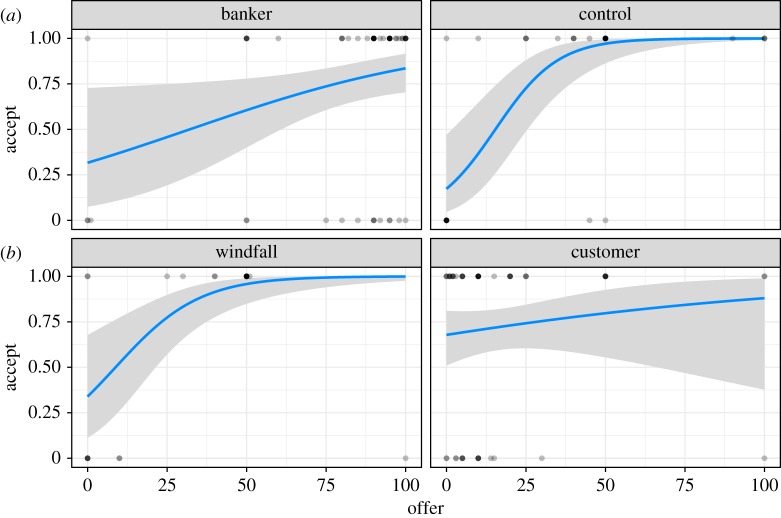
(*a*,*b*) Plot of logistic regression model of responders' acceptance probability as a function of offer amount and condition (grey shaded areas indicate 2 s.e., rug indicates offers). Model coefficients are in table 3.

**Figure 9.**
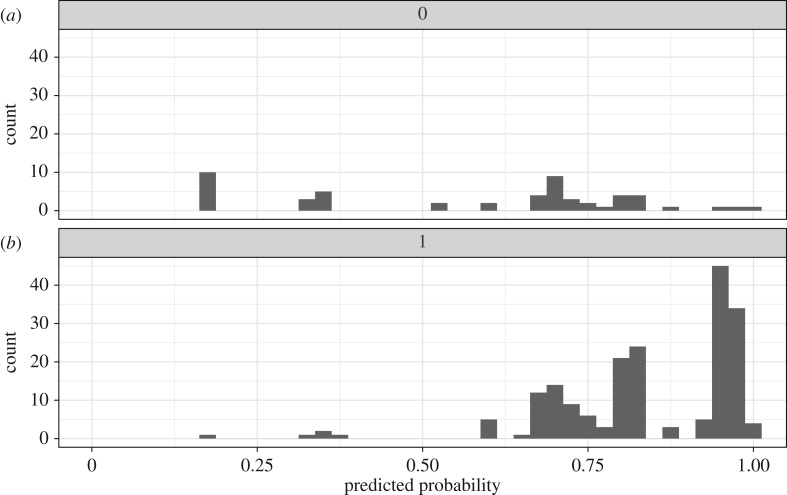
Plot of fitted probabilities of acceptance in the logistic regression model that includes experimental condition and offer amount (figure 8), for true rejections (*a*) and true acceptances (*b*). If the logistic regression model has perfect predictability, then true rejections should cluster at 0 and true acceptances should cluster at 1. This logistic regression model was better at predicting true acceptances (*b*) than true rejections (*a*). Tjur's coefficient of discrimination, *D* = 0.266, is the difference in the mean probabilities of true acceptances minus the mean probabilities of true rejections. This measure can be used as a logistic regression analogue to an *R*^2^ coefficient of determination [49].

**Figure 10.**
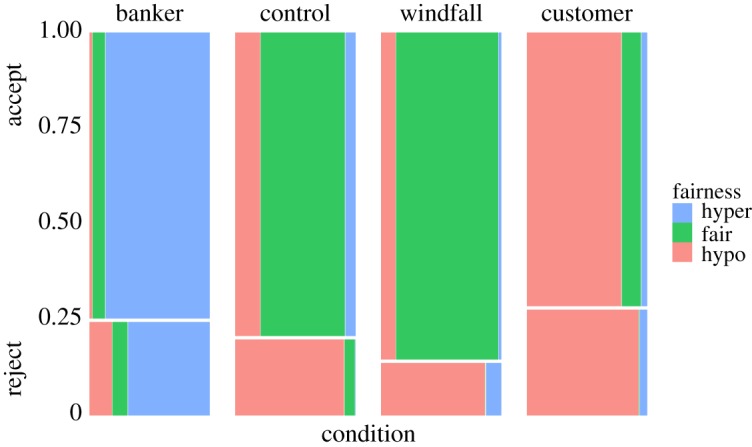
Mosaic plot of frequencies of responders' acceptance and rejection of offers under 50% (‘hypo’), at 50% (‘fair’), and over 50% (‘hyper’) by condition.

The probability of offer acceptance was modelled using a logistic regression (table 3 and figure 8). The probability of offer acceptance was low for small offers, and increased sharply in the control and windfall conditions as a function of offer amount. The customer and banker conditions had ‘flatter’ acceptance probabilities across offer amounts, although note that the few data points in the high/low offer ranges makes this a dubious distinction. Tjur's coefficient of discrimination, a pseudo *R*-squared, was *D* = 0.266. Inspection of the distribution of probabilities for true rejections (figure 9, top) and true acceptances (figure 9, bottom) indicated that our model was reasonably good at predicting acceptances but poor at predicting rejections. Overall, the responders' acceptance criteria aligned with our predictions: in the control and windfall conditions, responders largely accepted fair 50–50 splits with some tolerance for ‘hypo-fair’ (i.e. less than 50%) offers, but most of the latter were rejected. In the banker condition, 64.9% of participants offered 90% or more of their allocation, with modes at 90% (*n* = 13) and 95% (*n* = 12). When bankers were responders (in the customer condition), 68.6% accepted hypo-fair offers (those less than 50%). Customers in the responder role, on the other hand (in the banker condition), rejected 20.4% of hyper-fair offers (those greater than 50%). In the customer condition, most of the acceptances (and rejections) were hypo-fair, while most acceptances *and rejections* in the banker condition were ‘hyper-fair’ (i.e. greater than 50%) offers (figure 10).

**Table 3.** Logistic regression model of responder acceptance probability as a function of offer amount and condition. Coefficients are log odds. The base condition is the banker condition. Null deviance: 255 on 243 degrees of freedom. Residual deviance: 196 on 236 degrees of freedom. See figure 8 for an effects plot.

term	estimate	s.e.	statistic	*p*-value
intercept	−0.77	0.89	−0.86	0.39
conditioncontrol	−0.79	1.16	−0.69	0.49
conditionwindfall	0.10	1.15	0.09	0.93
conditioncustomer	1.51	0.96	1.57	0.12
offer	0.02	0.01	2.21	0.027
conditioncontrol:offer	0.08	0.03	2.93	0.0034
conditionwindfall:offer	0.05	0.02	2.18	0.029
conditioncustomer:offer	−0.01	0.02	−0.63	0.53

**Figure 11.**
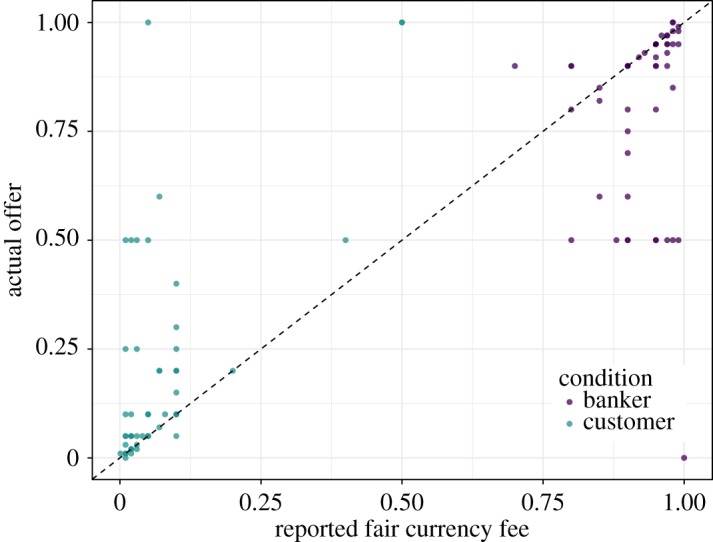
Amount offered (%) among proposers in the banker and customer conditions, as a function of their reported opinions about fair currency fee (%). Conditions are separately represented by colour.

The proposers' low offer amounts in the customer condition and high offer amounts in the banker condition appeared to be fairly consistent overall with their reported fair currency fees. In particular, most proposers in the customer condition both reported that a fair currency fee was a low percentage amount and offered low percentage amounts to the bankers. In the banker condition, most proposers both reported that a fair currency fee was a low percentage amount and offered high percentage amounts to the customers (i.e. kept a small bank fee—see figure 11).

This was true even though most participants reported only moderate familiarity with currency exchange. Pilot study results indicated that there was no meaningful difference in behaviour between high- and low-experience individuals (see tables S3 and S4 in the pilot study electronic supplementary material). We collected perceived fair fees from institutions that are similar to currency exchange (e.g. ATM fees, broker fees and service charges) because they might be intuited as conceptually similar to bank fees for currency exchange. In the final study, these amounts were all considered to be fair at less than 10% (ATM fees: mean = 2.66%, s.d. = 7.79%; broker fees: mean = 6.23%, s.d. = 10.1%; service charges: mean = 3.17%, s.d. = 8.33%; currency exchange fee: mean = 7.85%, s.d. = 15.5%). Taken together, this could suggest that individuals with little experience with currency exchange overcome their lack of exposure by conceptualizing this scenario as similar to other ‘banker–customer’ situations.

**Figure 12.**
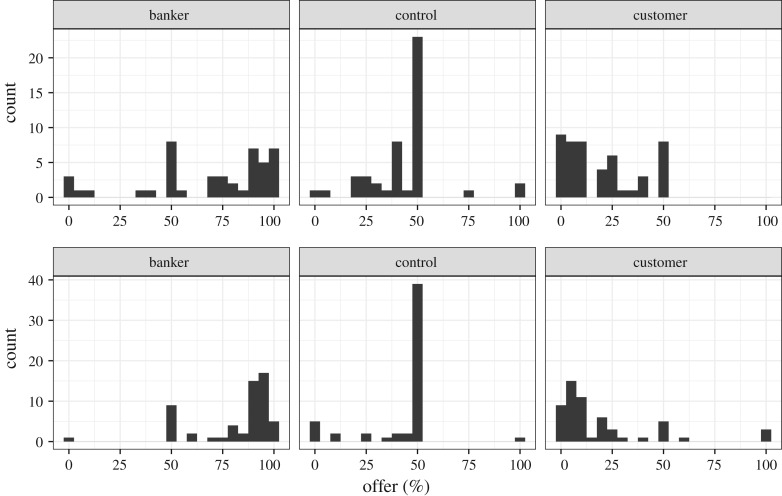
Distribution of offers in the pilot data (*a*) and final study (*b*). Study instructions were tweaked following the pilot study, which appeared to reduce low offers in the banker condition in the final study. The windfall condition is omitted in this figure because it did not have a corresponding pilot condition.

The pilot study results (previously discussed) informed our experimental design and caused us to slightly adjust our initial predictions and experimental protocol. To explore the effects of changing our protocol between pilot and experiment, we compare and contrast the results of this experiment to those of the pilot. As discussed in both this section and the ‘Pilot study’ section, we confirmed our predictions with large effect sizes between control and treatment conditions in both the pilot and the experiment. The offers in both (pilot and experiment) control conditions and both customer conditions did not significantly differ in central tendency (control condition: Mann–Whitney *U* = 1130, *p *= 0.39; customer condition: *U* = 1410, *p *= 0.66) or variance (control: Brown–Forsythe Levene *F* = 0.634, *p *= 0.43; customer: *F* = 2.7 × 10^−6^, *p *= 1). The banker condition in the experiment, however, yielded higher offers (*U* = 988, *p *= 0.067) with lower offer variance (*F* = 9.33, *p *= 0.0029) than the banker condition in our pilot. The latter had a significantly higher variance than the other conditions in the study, which as discussed, was probably an artefact of confusing instructions (see Pilot study). These comparisons indicate that the minor adjustments we made to study instructions following the pilot study improved our results (see figure 12 and electronic supplementary material for experimental GAMLSS analyses).

### 3.1. Exploratory analyses

We conducted exploratory analyses on both our pilot data and study data. Because participant attrition was a concern (Phase I electronic supplementary material and figure 5), we shortened the study by omitting exploratory items from the final study when analyses of the pilot data did not reveal strong evidence for theoretically important patterns. In particular, the SVO variable involved nine items, and was not part of any *a priori* hypothesis. In the pilot study, individuals classified as ‘prosocial’ according to their SVO score made slightly, but not significantly, larger offers across the three conditions, compared to individuals classified as ‘individualistic’. As noted in our discussion of pilot study results (Phase I electronic supplementary material), we would require a much larger sample size to have the power to detect an effect of SVO, if it exists. We therefore omitted the SVO items from the final study. To further shorten the study, we also omitted the minimum and maximum expected offers, which were not involved in any *a priori* hypothesis and had little theoretical significance.

We retained the other exploratory measures noted in the Methods. Not surprisingly, participants rated the fairness of the outcome significantly lower when offers were rejected than when offers were accepted (*M* = 2.4 versus *M* = 4.2 on a 1–5 point scale, *p *= 1.3 × 10^−33^ by Wilcoxon rank test). For responders, the expected payoffs appeared to influence accept/reject decisions. In general, when offers met or exceeded responders' expected payoffs, they were generally inclined to accept, rather than reject (*p *= 1.1 × 10^−4^). This effect was less distinct in treatment conditions relative to the control, but this appears to be an artefact of few actual offers below expected offers (i.e. with positive difference scores, where scores = expected payoff − actual offer). Recognition of the UG from previous experience did not have a significant effect on our key findings (*p *= 0.76). For details on these results, with additional exploratory analyses, see Phase II electronic supplementary material.

## 4. Discussion

Most studies of the UG provide the rules but little or no context for participants. We predicted that (i) in a UG that was unambiguously framed as a well-known economic institution (currency exchange), players would make and accept offers according to the norms of that frame, even if those offers deviated substantially from offers seen in unframed UGs, or from offers predicted by rational decision theory. Moreover, previous work suggested that generosity in the UG might in part be an artefact of low subjective value of windfall gains [50]. We therefore predicted that (ii) in a UG framed as a windfall (with the explicit expectation of fairness) there would be more 50–50 splits, and hence less variance, relative to the control condition.

**Figure 13.**
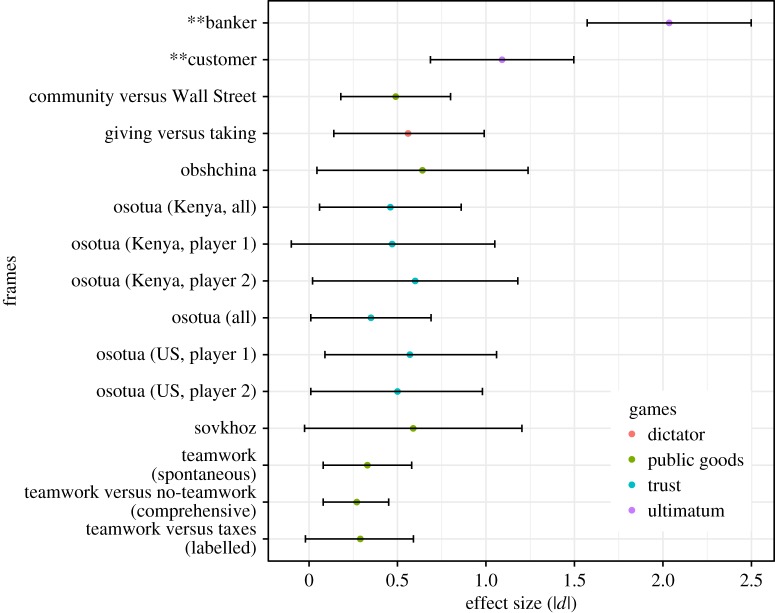
Effect sizes (absolute values of Cohen's *d*) from this study (denoted by ‘**’) and a representative sample of previous framing studies. Colours represent game type and bars indicate 95% CI. For reference, *d* = 0.8 is considered a large effect by convention.

Prediction (i) was confirmed: whereas the mean offer in the control condition was 42.1% (a value similar to that found in many other UG studies), proposers in the customer condition offered significantly smaller amounts than participants in the standard UG (control) condition (mean = 18.7%, *d* = 1.09, *p *< 0.001), and proposers in the banker condition offered significantly larger amounts than those in the control condition (mean = 81.8%, *d* = −2.04, *p *< 0.001). Note that the direction of the effect reversed when the roles were reversed (figures 6 and 7). To our knowledge, these framing effect sizes of *d* = 1.09 and *d* = −2.04 are among the largest found in the experimental framing literature (figure 13).

Moreover, our results confirm our predictions that in the banker condition, proposers would commonly make hyper-fair offers (greater than 80%) that would nevertheless suffer a non-trivial risk of rejection, whereas in the customer condition, proposers would commonly make hypo-fair offers (less than 20%) that would nevertheless enjoy high acceptance rates (figure 10—see also figures S2 and S3 in the electronic supplementary material for more detail). These are highly unusual patterns in the UG [8,51,52].

The results supporting prediction (i) are consistent with the observation that proposer behaviour can be manipulated by implicit asymmetries in entitlement to the stake [25,53]. In a typical currency exchange scenario, entitlement is rigidly defined: a customer is the proprietor of an initial sum, and the banker exchanges a service for a (usually small) bank fee. In general, well-defined institutional norms evolve culturally to facilitate decision-making in frequently encountered scenarios, reducing or eliminating the need for individuals to gather information and consider costs and benefits (in contrast to the idea that these norms provide a group benefit—see [14,54,55]). In our case, we expected our currency exchange vignettes to impress upon participants a mutually understood implication of customer entitlement to the sum, regardless of proposer role. Furthermore, we attribute the novel effect sizes to our use of cue-rich vignettes, used to unambiguously present specific, socially relevant frames of reference to participants.

Prediction (ii) was not confirmed. The offer variances in the windfall and control conditions were not significantly different, contrary to predictions. The windfall condition, relative to the control condition, yielded a higher percentage of 50–50 offers (85.2% versus 72.2%) and higher acceptance rates (83.6% versus 79.6%), however, which loosely supports Arkes *et al*. [50]. If the windfall condition could be considered a sort of ‘fairness ceiling’ in our study (addressing the question, ‘how fairly will proposers play when we ask them to play fairly?’), then it is possible that participants in the control will always be generous enough to ‘hover close to the ceiling’. On this view, it would not be unreasonable to consider our failure to confirm prediction (ii) to be a true negative result.

### 4.1. Conformance to, and deviance from, normative offers

We hypothesized that proposers in the currency exchange conditions would make offers conforming to their culturally acquired norms for currency exchange. However, different participants might have somewhat different views on the normative, or ‘fair’, offer in currency exchange. After the completion of the UG, we therefore asked all proposers to state what they felt was a ‘standard and fair’ fee for currency exchange. We then compared their actual offer to their stated fair fee. In the customer condition, 28.6% participants offered exactly their stated fair fee, and 73.2% offered within $0.10 of their fair fee. In the banker condition, 38.6% participants offered exactly their stated fair fee, and 70.2% offered within $0.10 of their fair fee. Hence, a majority of proposers offered amounts that were close to what they considered to be a fair fee.

The minority of proposers whose offers deviated more than $0.10 from their fair fee tended to offer amounts that were closer to a 50–50 split, which in the customer condition amounted to more generous offers, and in the banker condition amounted to stingier offers. Thus, even with a richly framed UG with normative offers near 0% and 100%, a substantial minority of participants still opted to make offers closer to the 50–50 split seen in studies of the unframed UG (exact 50–50 splits were *N* = 5 in customer condition, *N* = 9 in the banker condition; figure 11).

There are a number of possible reasons why participants' actual offers deviated from their stated fair fees. One group of possibilities involves mistakes, e.g. confusion about the instructions or lingering ambiguity about the frame [56–58]. The few offer(s) at the opposite end of each trend, for example (*N* = 3 offered 100% in customer condition, *N* = 1 offered 0% in banker condition), might have reflected some confusion. This may have also applied to some offers around a 50–50 split, particularly when the credibility of the experimental frame is low [59–61], or when the social relationship with the subject of interaction is not well defined [62].

Another group of possible explanations involves more ‘intentional’ (though not necessarily conscious) decisions to achieve some goal. For example, most AMT users aim to maximize income by quickly completing many low-paid tasks over a sustained period [63,64], and some might have played cooperatively by default (i.e. letting some kind of cooperative social-exchange heuristic override context-sensitive cues—see [65,66]). Fairness is often a safe bet for estimating the expectations of others when rules are uncertain and returns are contingent on offer approval [4,67]. Proposers might also prefer prosocial behaviour [68–71], which would help explain the striking similarity between offer distributions in our control and windfall conditions. Alternatively, some have argued that humans evolved other-regarding preferences. For discussion, see [72–75].

### 4.2. Comparison of final results to pilot data

Our pilot study (which was pre-registered with Open Science Framework: - osf.io), confirmed our *a priori* predictions about the effects of rich framing cues on offers in the UG. Nevertheless, our pilot study had more, and larger, deviations from our predicted offers than we expected. For the final study, we therefore modified our study instructions to reduce ambiguity about both the banker and customer conditions (see appendix of study materials in the electronic supplementary material). These modifications seem to have had their intended effect: in the pilot study, 35.8% of proposers in the banker condition made offers ≤50%, whereas in the final study only 17.2% made offers ≤50% (figure 12). The vignette-style approach in our final study allowed us to replicate the key findings in our instruction-style pilot study, but with larger effect sizes, higher conformity to institutionally defined values, and lower individual offer variation.

### 4.3. Excluded participants

In accordance with our *a priori* exclusion criteria, we excluded data from 38.7% of participants who made offers in the UG. Excluded participants either opted out of the study before it was completed or failed the attention check questions. Distributions of offers in the banker and customer conditions were similar for the included and excluded participants, and including data from the excluded participants had only a small effect on the regression coefficients that compare the customer and banker conditions to the control condition. The included participants in the control and windfall conditions, however, commonly offered 50%, whereas excluded participants in these conditions often offered 0%. We do not have a good explanation for the propensity of excluded participants to offer 0% in the control and windfall conditions, but it is worth noting that among the participants who opted out, rejections were exceedingly common. Overall, these differences between included and excluded participants had only a marginal impact on our main results. (See electronic supplementary material for more detailed analyses, with model summaries, ANOVA results and effects plots.)

## 5. Conclusion

Large offers in the UG challenge rational actor models of economic behaviour. One potential explanation is that the deliberate absence of contextual cues in the standard UG creates substantial ambiguity, leading participants to employ a variety of decision strategies (e.g. [5]). If so, rich contextual cues in a UG should reduce ambiguity, leading most participants to employ the same decision strategy. We framed the UG as a currency exchange scenario in which the proposer was a ‘customer’ and the responder was a ‘banker’ (the customer condition), and vice versa in a separate condition (the banker condition). As predicted, compared to offers in the unframed UG (*M* = 42.1%), participants in the customer condition made offers that were substantially and significantly smaller (*M* = 18.7%), which were usually accepted. Participants in the banker condition made offers that were substantially and significantly larger (*M* = 81.8%), which were occasionally rejected. The results reported here help confirm that experimental economic games are deeply rooted in situational and context-relevant interpretations [5,6,21,28,30]. The cognitively demanding task in these games therefore might not be utility maximization in some task environment (*sensu* [76]), but context identification prior to executing some associated heuristic(s).

Previous research has shown that mean offers in the UG and other games are different in different cultures (e.g. [9]), but conclusions drawn from correlations between unframed game outcomes and cultural characteristics are necessarily *ad hoc*. More importantly, the underlying mental process remains opaque to the researcher, and might not conform to traditional models of rational decision-making (e.g. unbounded rationality or optimization under constraints—see [15,77,78]). While our results provide further evidence for the importance of culturally transmitted norms in economic behaviour, the experimental research design of our study and other studies (e.g. [6,28,30]) go beyond observational designs to provide compelling evidence that change in an individual's frame of reference can *cause* dramatic change in economic behaviour.

The ‘cultural’ differences found in observational studies might therefore not be population-level cultural differences at all, but rather different people interpreting the unframed UG in different culturally appropriate ways. In fact, any UG scenario involving an agreed-upon split that is not 50–50 should focus attention on the perceived roles of participants involved. It is not clear that any large-scale/general inferences about human rationality—particularly in relation to social exchange behaviour—can be made based on results from unframed or decontextualized experimental economic games. Future research could help clarify the nature of decision-making as emergent from competing activations among a ‘pandemonium’ of context-relevant frames ([79,80]—see also [81,82]).

## Supplementary Material

Phase I supplementary materials (pilot results)

## Supplementary Material

Vignettes2

## Supplementary Material

Supplementary materials (Phase II)

## Data accessibility

Phase I pilot data: https://doi.org/10.5281/zenodo.1006724. Phase II final study data: https://doi.org/10.5281/zenodo.1006726. Phase II statistical analysis code: https://doi.org/10.5281/zenodo.1006730.
