# Correction to ‘Radical framing effects in the ultimatum game: the impact of explicit culturally transmitted frames on economic decision-making’

**DOI:** 10.1098/rsos.180120

**Published:** 2018-02-28

**Authors:** Aaron D. Lightner, Pat Barclay, Edward H. Hagen

*R. Soc. open sci.* **4**, 170543. (Published 20 December 2017). (doi:10.1098/rsos.170543)

The first sentence of §4.2 currently reads ‘Our pilot study (which was pre-registered with Open Science Framework: - osf.io),…’.

It should read: ‘Our pilot study (which was pre-registered with Open Science Framework: https://osf.io/qbp7h/),…’.

