# Supplementary material for: Radical framing effects in the ultimatum game: the impact of explicit culturally transmitted frames on economic decision-making
Source: R Soc Open Sci. 2017 Dec 20;4(12):170543. doi: 10.1098/rsos.170543 (PMC5749986; doi:10.1098/rsos.170543)
Supplement: Phase I supplementary materials (pilot results) [file rsos170543supp1.docx]

**Phase I supplementary materials (pilot results)**

Prior to analysis, 80 cases (21% of total) were excluded based on: (1) incorrect responses to an attention check question corresponding to the given UG instructions, (2) nonsensical responses in a question about minimum and maximum expected amounts (i.e., responses reporting higher minimum amounts than maximum) and (3) exceptionally fast completion times (HIT times were recorded and duration times < 90 seconds were excluded, because this was roughly the amount of time the experimenter required to quickly complete a practice run of the HIT). These exclusionary criteria were to ensure that our analyzed sample likely consisted of attentive participants. (Including the excluded cases did not change our results. See Figure S1.) Each treatment condition will be referred to by the proposers’ roles henceforth (i.e., the "customer" condition is the condition in which the proposer had the customer role and the banker had the responder role, and vice versa for the "banker" condition).


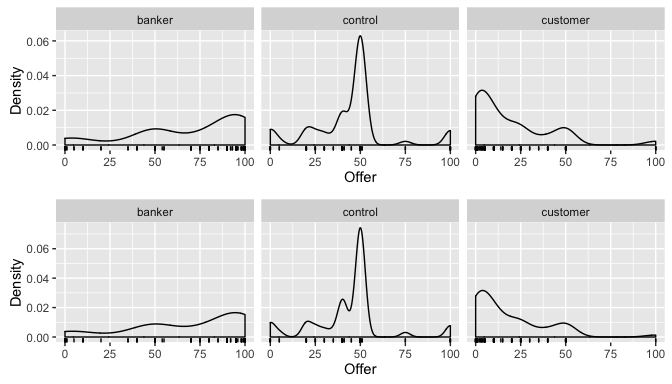


**Figure S1.** Density plots of all offers by condition, with raw data before exclusion (top row) and after exclusion (bottom row); bandwidth = 9.08, rug indicates offers.

**Offers Across Conditions**

The central tendency differences between treatment and control conditions were significant and conformed to our broader predictions (F = 97.04, p < 0.001, post hoc Tukey pairwise comparisons: p < 0.001 between customer-banker and customer-control conditions, p = 0.002 between banker-control conditions), with the banker condition (mean = 0.69, SD = 0.32) significantly higher than the control condition (mean = 0.435, SD = 0.205; t = -6.56, p < 0.001; Cohen’s D = -0.95) and the customer condition (mean = 0.18, SD = 0.205) significantly lower than the control condition (t = 8.75, p < 0.001; Cohen’s D = 1.24). See Table S1 and Figure S2.

| term | estimate | std.error | statistic | p.value |
| --- | --- | --- | --- | --- |
| (Intercept) | -0.262 | 0.206 | -1.274 | 0.203 |
| conditioncustomer | -1.252 | 0.329 | -3.808 | <0.001 |
| conditionbanker | 1.063 | 0.303 | 3.512 | <0.001 |

**Table S1.** Logistic regression of offers made by condition.


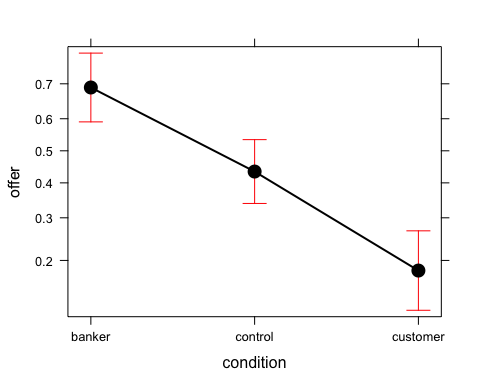


**Figure S2.** Generalized linear model of mean offers by condition.

Bars indicate +/- 2 SE. See text for details.

To be thorough and account for the deviations from normality in the offer distributions (Shipiro Wilk: banker W = 0.842, p < 0.001; customer W = 0.802, p < 0.001), nonparametric tests of central tendency change were also conducted using a Mann-Whitney U test (banker-control Z = -6.84, p < 0.001 with a banker median = 0.8 and control median = 0.5, control-customer Z = 8.24, p < 0.001 with a customer median = 0.1). Moreover, the predicted effect on banker-control and control-customer offers remained when offers were limited only to accepted offers (banker-control t = -6.93, p < 0.001; Z = -7.0, p < 0.001). The customer condition was associated with lower accepted offers when compared to those of the control condition (t = 8.93, p < 0.001; Z = 7.36, p < 0.001).

The probability of responder acceptance as a function of offer amount by condition was modeled by a logistic regression (Table S2, Figure S3) and Tjur’s coefficient of discrimination, a measure of fit, was D = 0.272 (Figure S4). The broader predictions made about responders' acceptance criteria were confirmed, in that participants mostly accepted fair offers and rejected "hypo-fair" offers (i.e., offers under a 50-50 split) in the control condition, mostly accepted hypo-fair offers in the customer condition, and rejected a substantial amount of "hyper-fair" offers (i.e., offers over a 50-50 split) in the banker condition (Figure S5).

| term | estimate | std.error | statistic | p.value |
| --- | --- | --- | --- | --- |
| (Intercept) | -4.793 | 2.726 | -1.758 | 0.079 |
| offer | 28.690 | 12.759 | 2.249 | 0.025 |
| conditioncustomer | 4.730 | 2.740 | 1.726 | 0.084 |
| conditionbanker | 4.642 | 2.774 | 1.673 | 0.094 |
| offer:conditioncustomer | -25.775 | 12.821 | -2.010 | 0.044 |
| offer:conditionbanker | -26.570 | 12.782 | -2.079 | 0.038 |

**Table S2.** Logistic regression of responder acceptance probability as a function

of offer amount by condition. Coefficients are log odds.


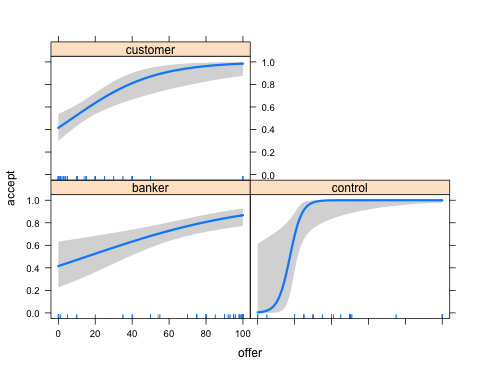


**Figure S3.** Plot of logistic regression of responders' acceptance probability as a function of offer amount by condition (gray shaded areas indicate 2 SE, rug indicates offers).


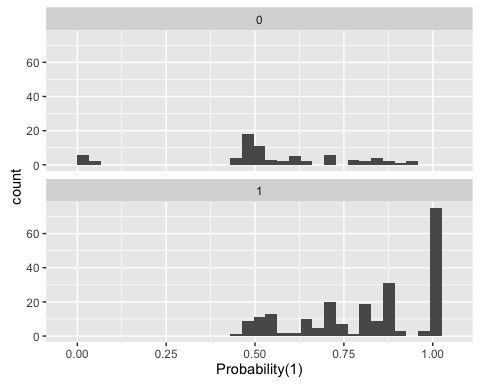


**Figure S4.** Plot of fitted probabilities of acceptance for true rejections (top) and true acceptances (bottom) as a function of experimental condition and offer amount. The coefficient of discrimination (Tjur's D) is the difference in the mean probabilities of true acceptances minus the mean probabilities of true rejections. This measure can be used as a logistic regression analogue to an R^2^ coefficient of determination (Tjur 2009).


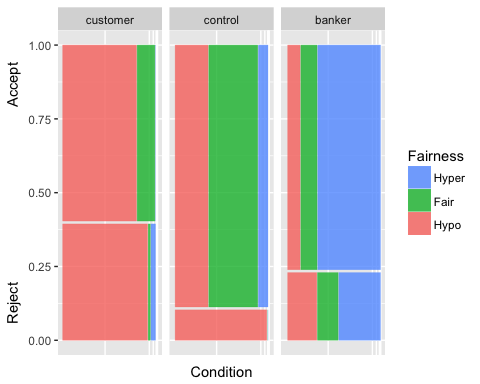


**Figure S5.** Mosaic plot depicting frequencies of responders' acceptance and rejection of offers under 50% ("Hypo"), at 50% ("Fair") and over 50% ("Hyper") by condition.

**Offer variances across conditions**

All tests for equality of variances were conducted with both Brown-Forsythe Levene-type tests and Fligner-Killeen tests, because both are robust to non-normal distributions to varying degrees. While the Bartlett’s test for homogeneity of variance was considered, it was ruled out because the treatment data were not normally distributed. The offer variances between control-customer conditions (control IQR = 0.1 and SD = 0.205, customer IQR = 0.225 and SD = 0.205) were not significantly different (Brown-Forsythe F = 0.676, p = 0.412; Fligner-Killeen chi-squared = 2.684, p = 0.101), and the variances between banker-control conditions (banker IQR = 0.455 and SD = 0.32) were contrary to our predictions, with significantly higher banker variation relative to the control (Brown-Forsythe F = 23.2, p < 0.001; Fligner-Killeen chi-squared = 26.3, p < 0.001). Because of this result and the Mann-Whitney U test assumption of equal variances, a two-sample Brown-Mood median test was used to re-check the significant central tendency difference between banker and control conditions, because it is a more appropriate and robust nonparametric test given unequal variances. This result remained significantly different, conforming to our predictions (Z = 7.47, p < 0.001). A Kolmogorov-Smirnov test was also used to check for any differences in the distributions between banker and control conditions (D = 0.559, p < 0.001).

To test our predictions of a moderating effect of currency exchange experience on offer variation, we created an additive experience variable (which will be referred to henceforth as “forex,” a sum of international travel and currency exchange experience variables) from the Likert scores in currency exchange experience-related questions. We then compared offer distributions of high experience individuals (upper forex quartile) with those of low experience individuals (lower forex quartile). The offer variation in each experience level of the customer condition (high experience median = 0.1, IQR = 0.255; low experience median = 0.1, IQR = 0.215) was not significantly different from that of its corresponding level in the control condition (high experience median = 0.5, IQR = 0.1; low experience median = 0.5, IQR = 0.163), and this same null result applied to offer variation in each experience level of the banker condition (high experience median = 0.9, IQR = 0.115; low experience median = 0.775, IQR = 0.268) and corresponding experience levels in the control condition. Thus, adding experience with currency exchange as a parameter (AIC = 203.6) into our condition-only model (AIC = 191.3) did not improve its fit (Tables S3 and S4; $\Delta$AIC = 12.17), indicating that this aspect of our predictions was not confirmed. Separated offer distributions by high and low forex levels are shown in Figure S6.

| term | estimate | std.error | statistic | p.value |
| --- | --- | --- | --- | --- |
| (Intercept) | 1.033 | 0.257 | 4.013 | 0.000 |
| conditioncontrol | -1.219 | 0.392 | -3.108 | 0.002 |
| conditioncustomer | -2.722 | 0.399 | -6.829 | 0.000 |

**Table S3.** Logistic regression of offer amount by condition only

(NA responses in forex variable excluded, banker as reference condition).

| term | estimate | std.error | statistic | p.value |
| --- | --- | --- | --- | --- |
| (Intercept) | 0.975 | 0.718 | 1.358 | 0.175 |
| forex_exp | 0.193 | 0.333 | 0.579 | 0.563 |
| conditioncontrol | -0.970 | 0.964 | -1.006 | 0.314 |
| conditioncustomer | -2.723 | 1.102 | -2.471 | 0.013 |
| travel_exp | -0.190 | 0.308 | -0.619 | 0.536 |
| forex_exp:conditioncontrol | -0.237 | 0.477 | -0.496 | 0.620 |
| forex_exp:conditioncustomer | -0.270 | 0.484 | -0.558 | 0.577 |
| conditioncontrol:travel_exp | 0.156 | 0.441 | 0.354 | 0.724 |
| conditioncustomer:travel_exp | 0.298 | 0.465 | 0.640 | 0.522 |

**Table S4.** Logistic regression of offer amount by condition

and forex level, (banker as reference condition).


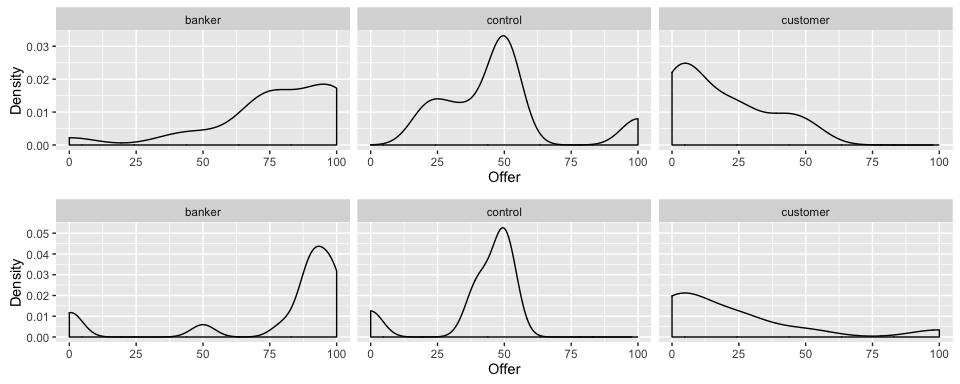


**Figure S6.** Density plot of offers among low forex (top row)

and high forex participants (bottom row).

While separating the offer distributions into high and low experience levels seemed to "narrow" the variation around the median offers (as shown in Figure S9), the variances between experience levels within conditions were not significantly different. To be certain, a KS test was used to detect any differences in offer distributions between low and high forex levels (banker D = 0.3, p = 0.453; customer D = 0.177, p = 0.983; control D = 0.187, p = 0.996). These distributions were not significantly different between experience levels, and thus indicate that this aspect of our predictions was not confirmed.

**Exploratory analyses**

For exploratory analyses, we asked participants to complete a social value orientation (SVO) survey, a measurement of prosocial preferences which categorized people into prosocial (N = 173), individualistic (N = 88) and competitive (N = 3) categories based on their responses. Prosociality had a biasing effect on offers in the control condition toward an equal split (prosocial mean = 0.444, SD = 0.205, median = 0.5, IQR = 0.1; individualistic mean = 0.365, SD = 0.162, median = 0.4, IQR = 0.3), the customer condition showed slightly more generous hypo-fair offers (prosocial mean = 0.192, SD = 0.225, median = 0.1, IQR = 0.235; individualistic mean = 0.134, SD = 0.138, median = 0.1, IQR = 0.24) and the banker condition showed more hyper-fair offers among prosocial participants, as opposed to more even splits among individualistic participants (prosocial mean = 0.776, SD = 0.26, median = 0.9, IQR = 0.48; individualistic mean = 0.541, SD = 0.326 median = 0.5, IQR = 0.45). While this trend was worthy of note, the overlapping error bars (Figure S7) suggest that an effect associated with SVO (if it exists) would require a higher powered study to be detected.


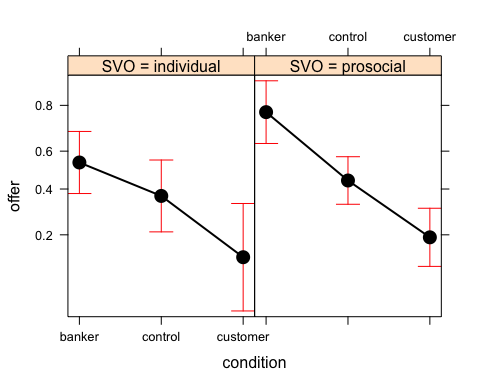


**Figure S7.** Generalized linear model of offers (mean and standard error) by condition and by SVO categorization (competitive excluded due to small sample size).

We also asked participants in the treatment conditions at the end of the survey if they recognized the game as a UG from previous experiences, and positive responses to this question (N = 16) appeared to have inflated some of the proposers' fair offers in the treatment conditions (Figure S8). Finally, we note a disconnect between game expectations, behavior and "real life" expectations, which was particularly substantial in the treatment conditions. For proposers in all conditions, actual offers made were lower than their self-reported "fair amount" in the experiment (termed "fair bank fee" in the treatment conditions), and this effect was particularly noticeable in the treatment conditions (Figure S9). Furthermore, all participants were asked what amount they considered to be a fair bank fee "in real life," and this did not seem to reflect the fair bank fee that they reported in the actual experiment (Figure S10). Finally, despite the lack of effect of forex level on offer variance, mixture models of offer distributions were created for additional exploratory insight using expectation maximization with GAMLSS in R. In particular, we established that the distribution in each treatment condition consisted of distinct constitutive distributions, which may reflect different subpopulations interpreting the game in one of two or three ways (discussed in more detail below; also see Figures S11 and S12).


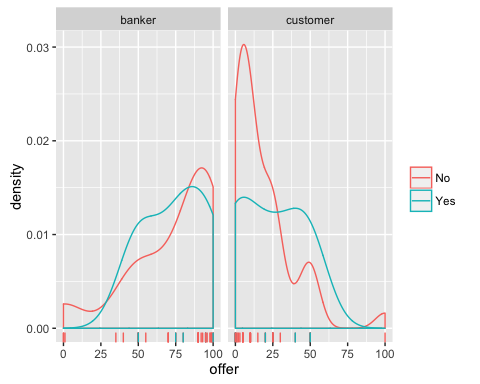


**Figure S8.** Density plot of proposers' offers by treatment condition and self-reported prior experience with and recognition of the UG (n = 18). Individuals are represented by color corresponding to those who answered "yes" or "no" to a question about their recognition of the game they played.


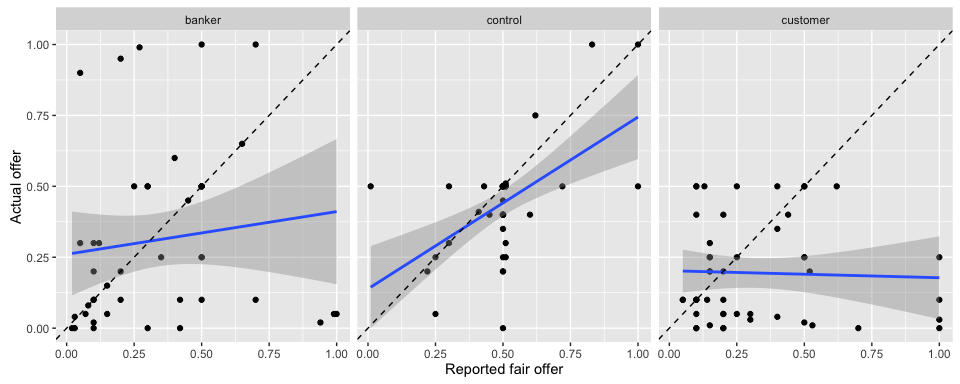


**Figure S9.** Actual offers made by proposers as a function of self-reported fair offers in the experiment, by condition (banker data were transformed to reflect a self-reported fair offer based on their "fair bank fee" response). The dashed line represents perfect 1:1 consistency between behavior and opinions about fairness in the experiment.


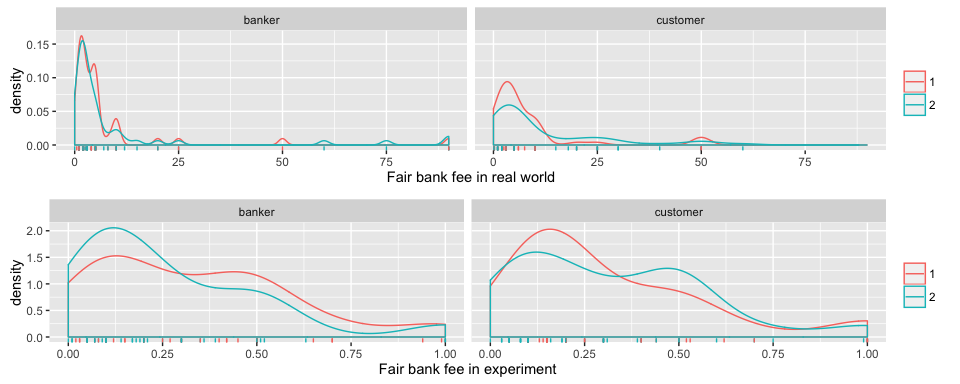


**Figure S10.** Treatment responses to the question about what was considered a fair bank fee in real world scenarios (top row) and what was considered a fair amount (termed "fair bank fee") in the experiment (bottom row). Colors are by Player ID (1 = proposer, 2 = responder).


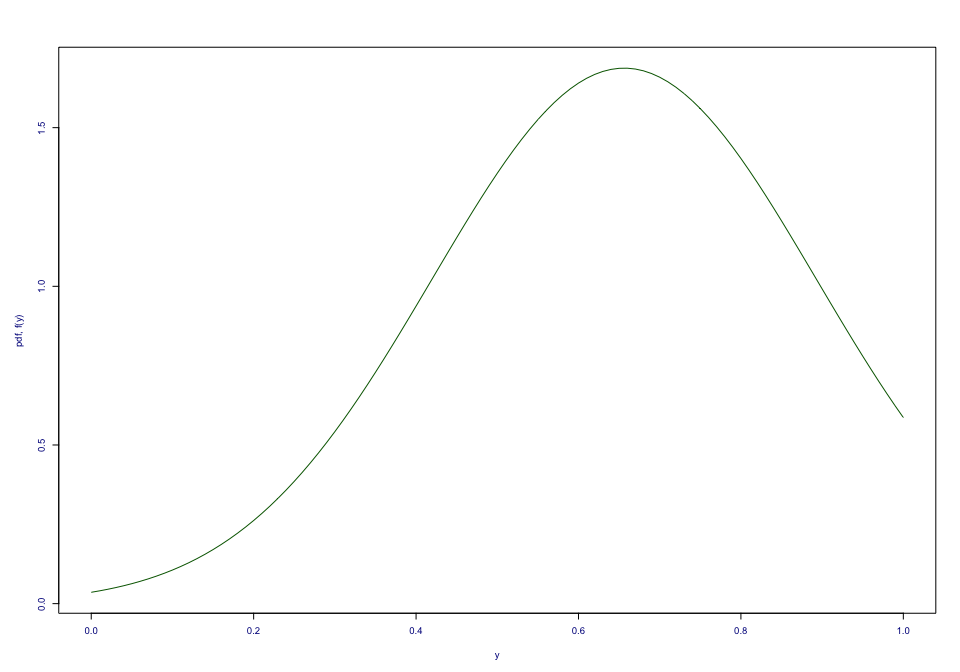

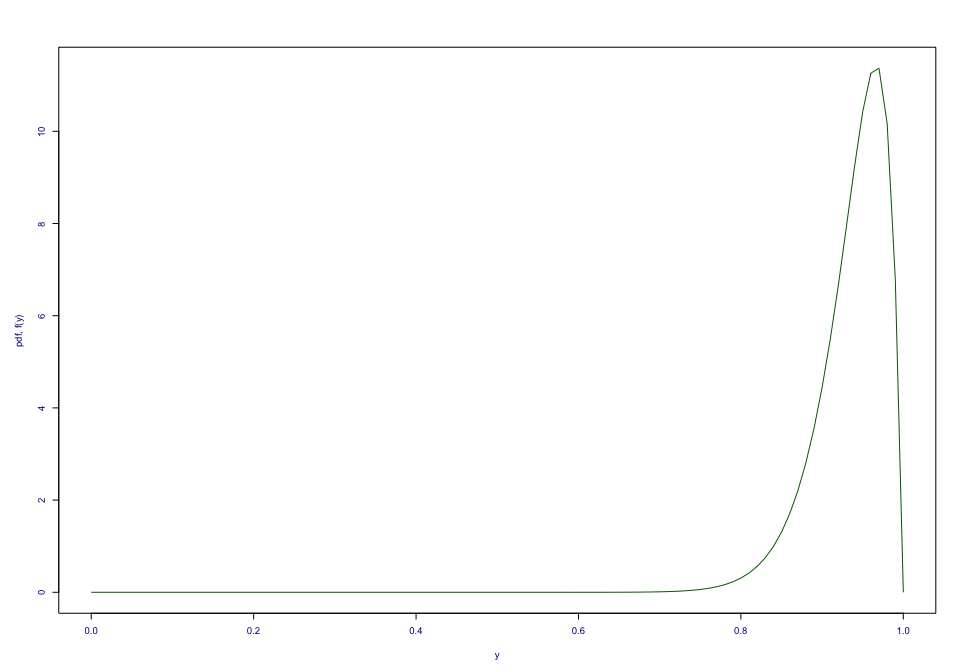

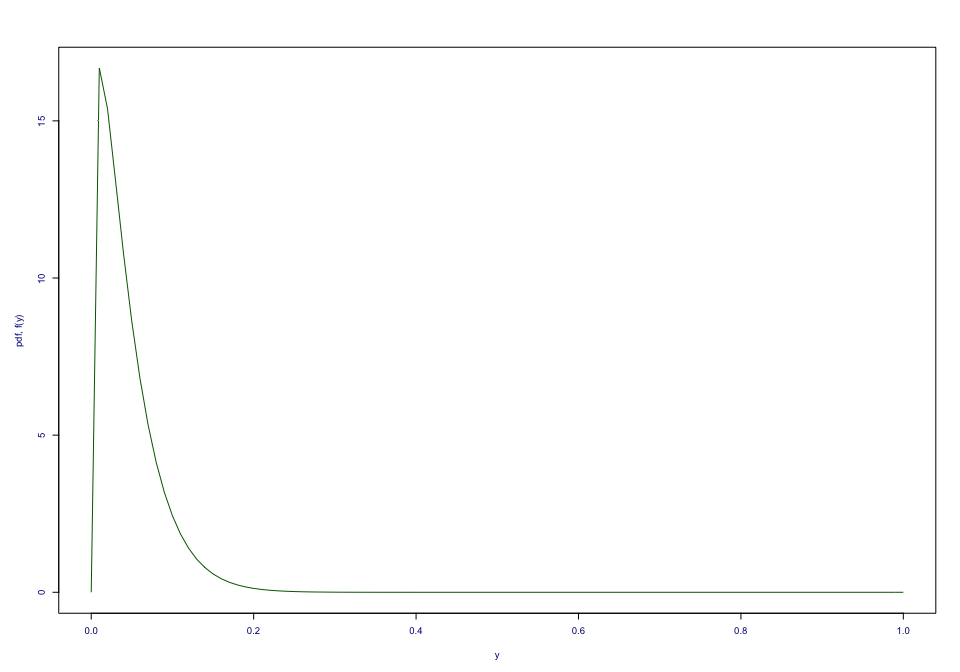


**Figure S11.** Mixture model for offer distribution in banker condition using expectation maximization to determine 3 constitutive distributions (top-bottom: normal: mu = 0.656, sigma = 0.236; inflated beta: mu = 0.937, sigma = 0.175, nu = 3.561×10^-8^, tau = 2.7610×10^-8^; inflated beta: mu = 0.0438, sigma = 0.182, nu = 9.6910×10^-8^, tau = 7.5110×10^-8^).


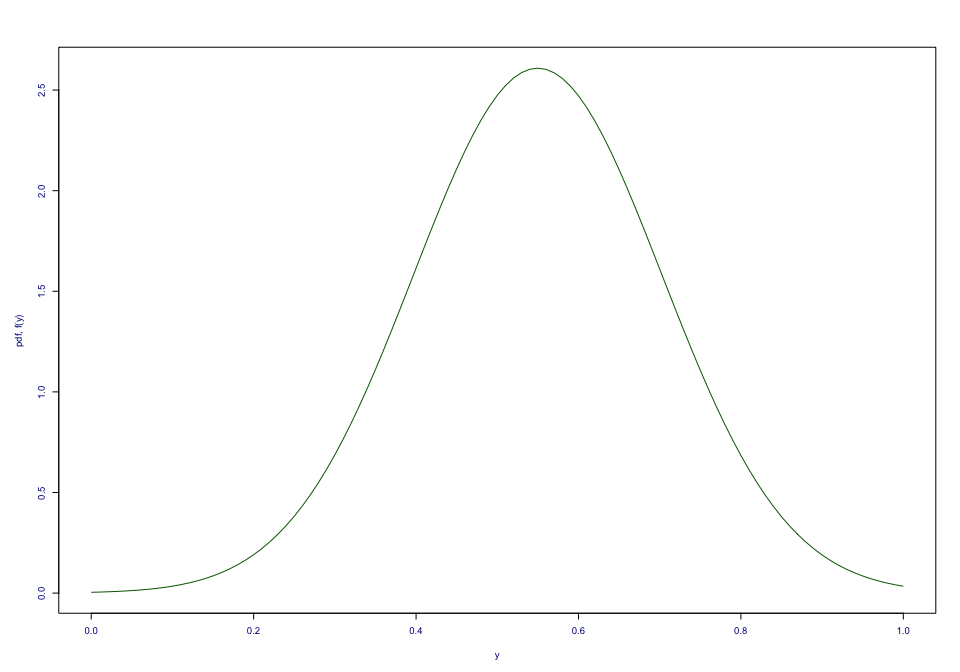

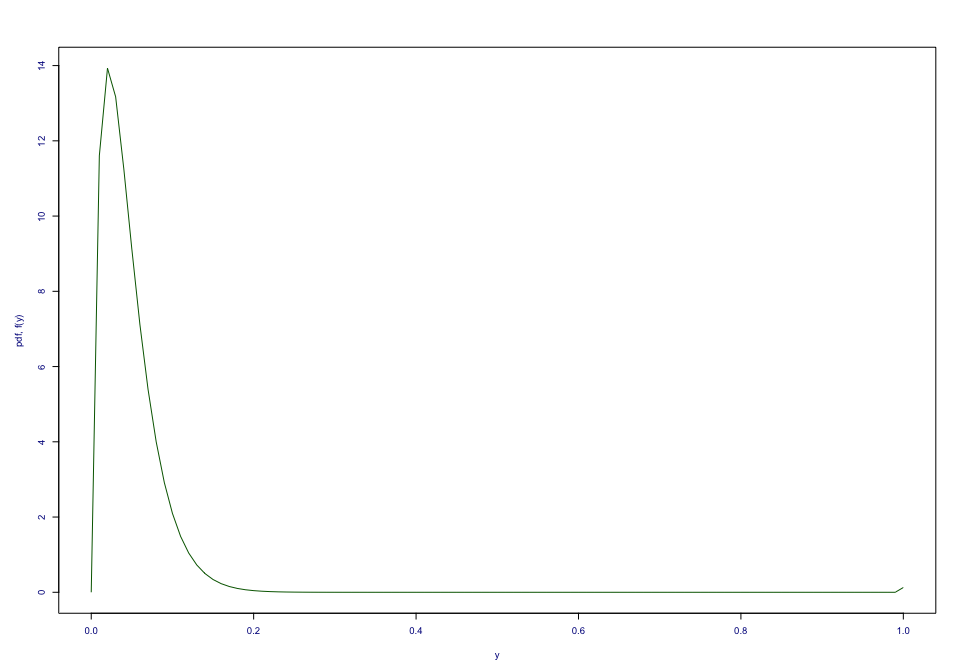


**Figure S12.** Mixture model for offer distribution in customer condition using expectation maximization to determine 2 constitutive distributions (top-bottom: normal: mu = 0.55, sigma = 0.153; inflated beta: mu = 0.0449, sigma = 0.155, nu = 10^-8^, tau = 0.145).
