# Supplementary material for: Radical framing effects in the ultimatum game: the impact of explicit culturally transmitted frames on economic decision-making
Source: R Soc Open Sci. 2017 Dec 20;4(12):170543. doi: 10.1098/rsos.170543 (PMC5749986; doi:10.1098/rsos.170543)
Supplement: Vignettes2 [file rsos170543supp2.docx]

**Banker vignette (proposer)**

In this study you are a banker at a foreign currency exchange service at an international airport. A traveler, who is another participant from mturk, approaches your desk to convert 100 virtual dollars (VD) into US dollars (USD). The currency exchange rate is 1 VD = 0.01 USD.

You can request a bank fee for providing the currency exchange services, as a percentage to keep out of the 100 VD. The traveler can accept or reject the bank fee.

If the traveler accepts your fee, then you will complete the currency exchange transaction and the VD will be converted to USD. You will keep the fee and the traveler will keep the USD minus your fee. If the traveler rejects your fee, no transaction will take place, and the currency will not be exchanged into US dollars.

At the end of the experiment, all VD will lose their value.

**Customer vignette (proposer)**

In this study you are an international traveler carrying 100 virtual dollars (VD) through an airport. You need to convert this money into US dollars (USD). You approach a banker at a currency exchange booth. The banker, who is another participant from mturk, informs you that the currency exchange rate from VD to USD is 1 VD = 0.01 USD.

You can offer the banker a percentage of the 100 VD as a bank fee for currency exchange services. The banker can accept or reject your proposed fee.

If the banker accepts your fee, then the money will be converted from VD to USD and you will leave the airport with real money in US dollars, minus the bank fee. If the banker rejects your fee, then no transaction will take place, and the currency will not be exchanged into US dollars.

At the end of the experiment, all VD will lose their value.

**Gift/ windfall vignette (proposer)**

You and another person on Mturk have both been selected to share a $1.00 bonus as a gift for completing this HIT. You are being given the gift in full, and we ask that you split it fairly with the other person, who is currently also completing this HIT.

You will make this split by entering the amount you will share with the other person, and he or she can accept or reject the portion that you chose to share.

If the other person accepts the portion, then you will both keep your shares of the bonus gift for completing the HIT. If the other person rejects the portion then neither you nor the other person will receive a bonus for this HIT.

**Banker vignette (responder)**

In this study you are a banker at a foreign currency exchange service at an international airport. A traveler, who is another participant from mturk, approaches your desk to convert 100 virtual dollars (VD) into US dollars (USD). The currency exchange rate is 1 VD = 0.01 USD.

The traveler will offer you a bank fee for providing the currency exchange services, as a percentage to keep out of the 100 VD. You can accept or reject the bank fee.

If you accept the fee, then you will complete the currency exchange transaction and the VD will be converted to USD. You will keep the fee and the traveler will keep the USD minus your fee. If you reject the fee, no transaction will take place, and the currency will not be exchanged into US dollars.

At the end of the experiment, all VD will lose their value.

**Customer vignette (responder)**

In this study you are an international traveler carrying 100 virtual dollars (VD) through an airport. You need to convert this money into US dollars (USD). You approach a banker at a currency exchange booth. The banker, who is another participant from mturk, informs you that the currency exchange rate from VD to USD is 1 VD = 0.01 USD.

The banker will request a percentage of the 100 VD as a bank fee for currency exchange services. You can accept or reject this proposed fee.

If you accept this fee, then the money will be converted from VD to USD and you will leave the airport with real money in US dollars, minus the bank fee. If you reject the fee, then no transaction will take place, and the currency will not be exchanged into US dollars.

At the end of the experiment, all VD will lose their value.

**Gift/ windfall vignette (responder)**

You and another person on Mturk have both been selected to share a $1.00 bonus as a gift for completing this HIT. The other person, who is currently also completing this HIT, is being given the gift in full. We are asking that he or she splits it fairly with you.

The other person will make this split by entering the amount s/he will share with you, and you can accept or reject the portion that is shared.

If you accept the portion, then you will both keep your shares of the bonus gift for completing the HIT. If you reject the portion, then neither you nor the other person will receive a bonus for this HIT.
