# Supplementary material for: Radical framing effects in the ultimatum game: the impact of explicit culturally transmitted frames on economic decision-making
Source: R Soc Open Sci. 2017 Dec 20;4(12):170543. doi: 10.1098/rsos.170543 (PMC5749986; doi:10.1098/rsos.170543)
Supplement: Supplementary materials (Phase II) [file rsos170543supp3.docx]

# Supplementary materials (Phase II)

Using our *a priori* exclusion criteria, we omitted data from 38.7% of participants who made an offer in the UG. It is therefore important to assess how the excluded offers differed from the included offers. Figure 6 depicts the distributions of offers of excluded vs. included participants, revealing a large difference between them only in the control and windfall conditions: 79% of the included participants offered 50% and only 7% offered 0%; in contrast, 21% of excluded participants offered 50% and 61% offered 0%).

These differences had only a marginal impact on our main results. We fitted a model of offers by condition that included all participants (N = 770 instead of N = 472). Results were nearly identical to those depicted in Table 2 and Figure 7. We also fitted a model with an additional variable that indicated excluded status. The main effect of condition was still highly significant, there was no significant main effect of exclusion on offers, and there was only a marginally significant exclusion X condition interaction (p = 0.078), probably due to the windfall condition (see Figure S1). We do not have a good explanation for the propensity of excluded participants to offer 0% in the control and windfall conditions. Many control and windfall participants who opted out immediately after the game/before the survey had low offers and high rejection rates (78.3% rejected, mean = 19.6%), and most others were excluded for their failure to pass attention checks.


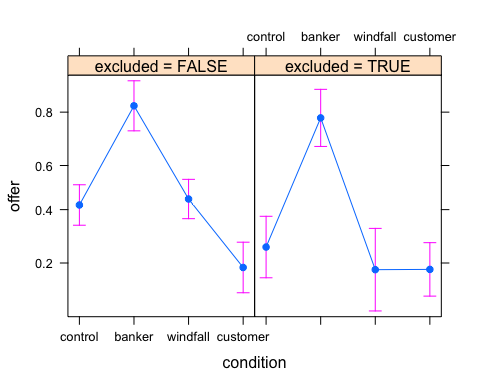


**Figure S1.** Generalized linear model of offers by condition and included vs. excluded status. Error bars indicate +/- 2 SE.

Table S1. ANOVA results for condition X excluded results.

| term | LR.Chisq | df | p.value |
| --- | --- | --- | --- |
| condition | 105.48 | 3 | ${10}^{-22}$ |
| excluded | 5.26 | 1 | $0.022$ |
| condition:excluded | 6.83 | 3 | $0.078$ |

Table S2. Summary results for condition X excluded results.

| term | estimate | std.error | statistic | p.value |
| --- | --- | --- | --- | --- |
| (Intercept) | -0.32 | 0.19 | -1.69 | $0.091$ |
| conditionbanker | 1.82 | 0.30 | 6.04 | $1.5\times{10}^{-9}$ |
| conditionwindfall | 0.11 | 0.26 | 0.42 | $0.68$ |
| conditioncustomer | -1.15 | 0.30 | -3.78 | $1.6\times{10}^{-4}$ |
| excludedTRUE | -0.77 | 0.35 | -2.24 | $0.025$ |
| conditionbanker:excludedTRUE | 0.55 | 0.50 | 1.11 | $0.27$ |
| conditionwindfall:excludedTRUE | -0.53 | 0.55 | -0.96 | $0.34$ |
| conditioncustomer:excludedTRUE | 0.74 | 0.49 | 1.51 | $0.13$ |


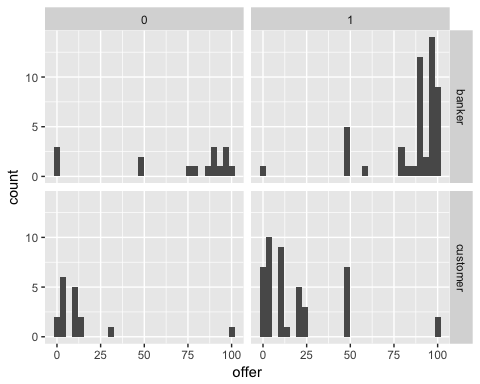


**Figure S2.** Histograms of offer amounts in the currency exchange treatment conditions by condition and responders' acceptance status (1 = accepted, 0 = rejected).


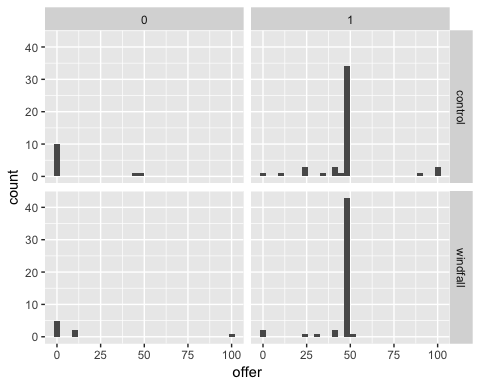


**Figure S3.** Histograms of offer amounts in the control and windfall conditions by condition and responders' acceptance status (1 = accepted, 0 = rejected).

## Exploratory measures

Responders' expected payoffs influenced their probability of acceptance, such that offers exceeding expectations (expectation - offer < 0) were more readily accepted than offers below expectations (expectation - offer > 0). This trend was particularly distinct in the control condition, though indistinct trends in the treatment conditions are limited by the fewer offers below expectations (Table S3 and Figure S4). A potentially useful diagnostic measure was a survey question asking participants if they recognized the game as a bargaining game from previous experience. Offer trends among "yes" and "no" responses were not significantly different (Figure S7). Overall, 14.8% of participants recognized the UG, and this number was < 25% in all experimental conditions and among proposers and responders (Figure S8). We also measured expectations with a survey question asking what participants expected to receive as a payoff (Figure S9), though it is not clear whether the participants interpreted this question as either total payoff (UG earnings + $0.25 participation fee) or UG earnings alone. Perhaps more importantly, we asked participants if they thought that the outcome of the experiment was fair. Any glaringly high rates of dissatisfaction after, e.g., a treatment condition or solely among proposers or responders could have reflected any unfair framing practices or unintentional confusion in our vignette approach. We find that participants were not dissatisfied on average across roles any of the control nor treatment conditions, nor were the proposers or responders uniquely dissatisfied in any particular condition (Figure S10, Table S5).

Table S3. Logistic regression model of difference scores (expected offer minus actual offer) among responders by condition. Estimates are log odds. The base condition is the control condition. See Figure S4 for an effects plot.

| term | estimate | std.error | statistic | p.value |
| --- | --- | --- | --- | --- |
| (Intercept) | 2.45 | 0.57 | 4.29 | $1.8\times{10}^{-5}$ |
| diff_score | -6.28 | 1.83 | -3.44 | $5.8\times{10}^{-4}$ |
| conditionbanker | -1.35 | 0.64 | -2.10 | $0.036$ |
| conditioncustomer | -1.17 | 0.67 | -1.73 | $0.083$ |
| conditionwindfall | -0.12 | 0.76 | -0.15 | $0.88$ |
| diff_score:conditionbanker | 6.08 | 2.00 | 3.04 | $0.0024$ |
| diff_score:conditioncustomer | 4.76 | 2.00 | 2.38 | $0.017$ |
| diff_score:conditionwindfall | 2.30 | 2.43 | 0.95 | $0.34$ |


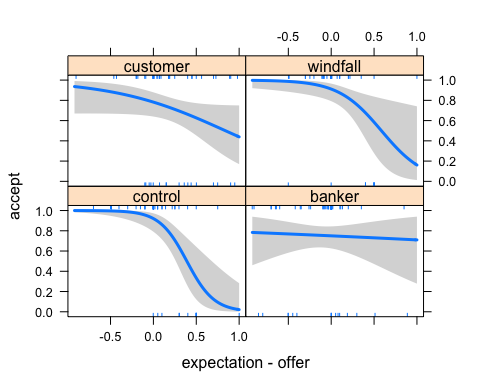


**Figure S4.** Plot of logistic regression model of responders' acceptance probability as a function of difference scores (expected offer minus actual offer) among responders by condition (gray shaded areas indicate 2 SE, rug indicates offers). Model coefficients are in Table S3.


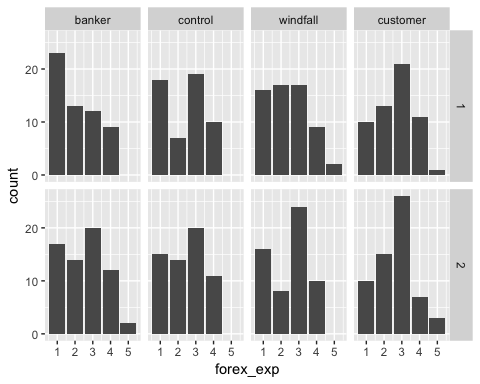


**Figure S5.** Likert scale responses to level of experience (1 = no experience at all, 5 = extensive experience) with currency conversion by condition and role (1 = proposer, 2 = responder).


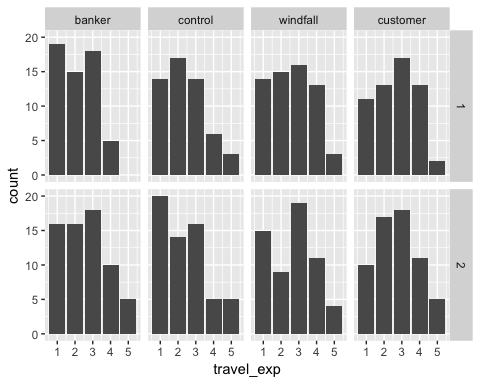


**Figure S6.** Likert scale responses to level of experience (1 = no experience at all, 5 = extensive experience) with international travel by condition and role (1 = proposer, 2 = responder).

Table S4. Logistic regression model of mean offers by condition and recognition of the UG from previous experience. Estimates are log odds. The base condition is the control condition. See Figure S7 for an effects plot.

| term | estimate | std.error | statistic | p.value |
| --- | --- | --- | --- | --- |
| (Intercept) | 2.45 | 0.57 | 4.29 | $1.8\times{10}^{-5}$ |
| diff_score | -6.28 | 1.83 | -3.44 | $5.8\times{10}^{-4}$ |
| conditionbanker | -1.35 | 0.64 | -2.10 | $0.036$ |
| conditioncustomer | -1.17 | 0.67 | -1.73 | $0.083$ |
| conditionwindfall | -0.12 | 0.76 | -0.15 | $0.88$ |
| diff_score:conditionbanker | 6.08 | 2.00 | 3.04 | $0.0024$ |
| diff_score:conditioncustomer | 4.76 | 2.00 | 2.38 | $0.017$ |
| diff_score:conditionwindfall | 2.30 | 2.43 | 0.95 | $0.34$ |


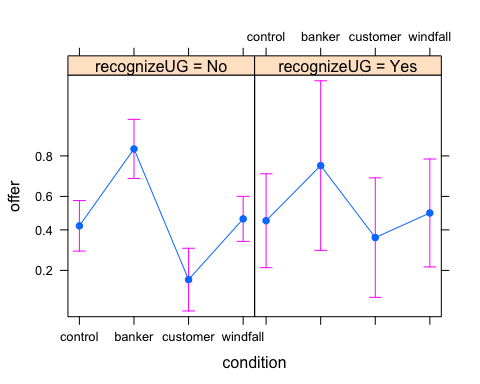


**Figure S7.** Effect plot of the mean offer (as a proportion of the maximum possible offer) by condition and recognition of the UG. Bars indicate +/- 2 SE. Model coefficients are in Table S4. See text for details.


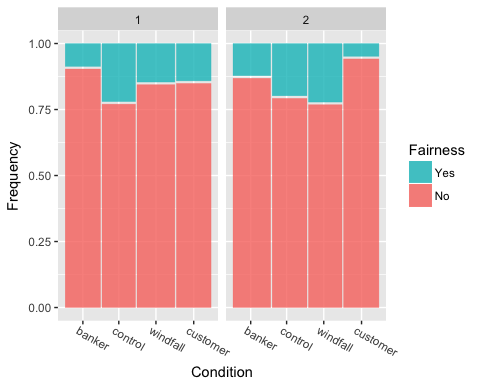


**Figure S8.** Frequencies of "yes" vs. "no" responses to the question about whether or not participants recognized the UG from previous experience, by condition and role (1 = proposer, 2 = responder).


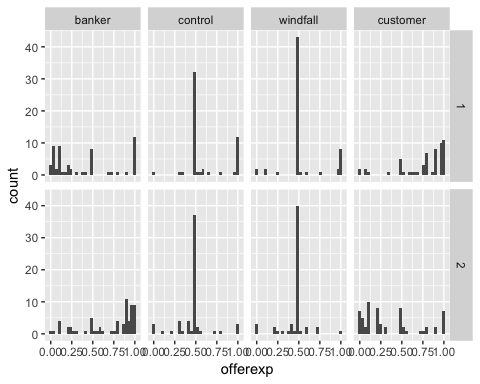


**Figure S9.** Histogram of responses to the question about what participants expected to receive as a payoff in the task they completed, by condition and role (1 = proposer, 2 = responder).


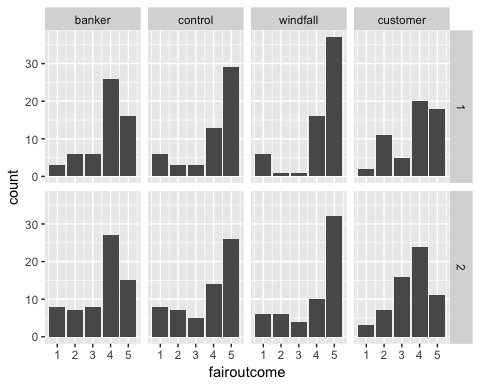


**Figure S10.** Likert scale responses to the statement that participants thought that the outcome of the game was fair (1 = strongly disagree, 5 = strongly agree) by condition and role (1 = proposer, 2 = responder).

Table S5. Summary results for participant opinions about fairness of game outcomes.

| Player type | condition | Mean | SD |
| --- | --- | --- | --- |
| Proposer | banker | 3.81 | 1.13 |
| Proposer | control | 4.04 | 1.36 |
| Proposer | windfall | 4.26 | 1.24 |
| Proposer | customer | 3.73 | 1.21 |
| Responder | banker | 3.52 | 1.30 |
| Responder | control | 3.72 | 1.46 |
| Responder | windfall | 3.97 | 1.41 |
| Responder | customer | 3.54 | 1.07 |
| Proposer | All | 3.96 | 1.24 |
| Responder | All | 3.68 | 1.32 |
| All | All | 3.82 | 1.29 |

In the pilot study, we used our GAMLSS analyses as a diagnostic to construct mixture models in our widely varying treatment distributions. To be thorough, we include these for the experimental study and note that both treatment conditions consist only of two distribution clusters rather than the problematic three seen in the pilot study (each around our predicted values and a 50-50 offer - see Figures S11 and S12). This is also apparent in the Results section (Figure 11), and we discuss these particular deviations from our predictions at some length in the Discussion section.


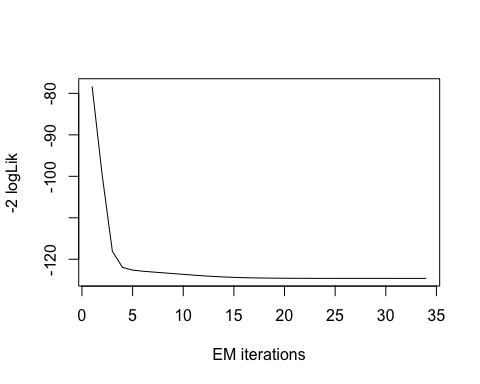


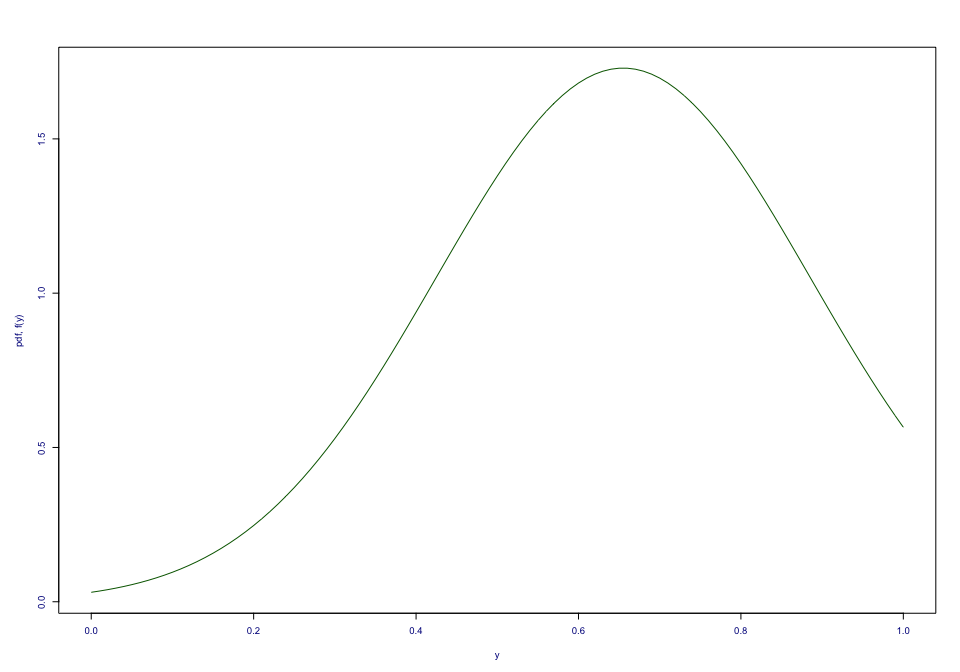

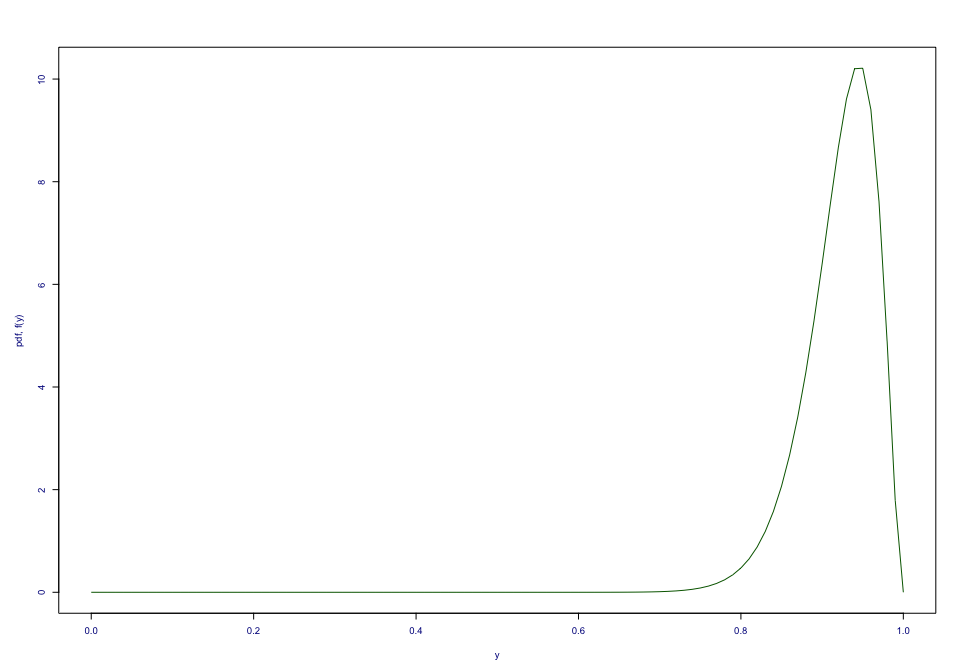


**Figure S11.** Mixture model for offer distribution in banker condition using expectation maximization to determine 2 constitutive distributions (top-bottom: normal: mu = 0.655, sigma = 0.231; inflated beta: mu = 0.922, sigma = 0.161, nu = $3.41\times{10}^{-6}$, tau = 9.7710^{-9}).


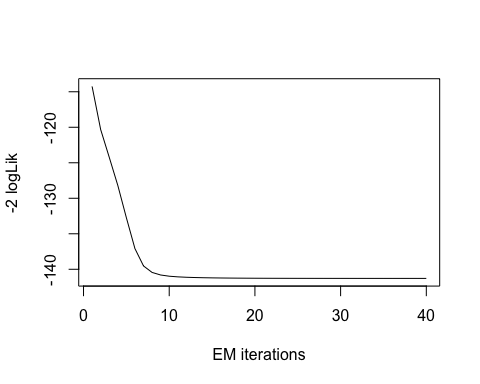


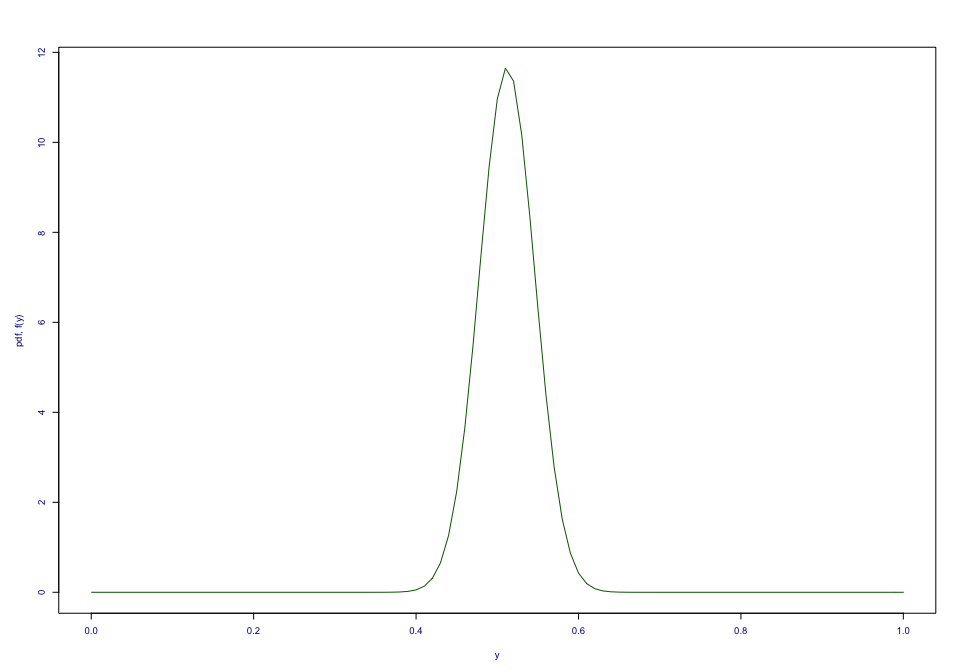

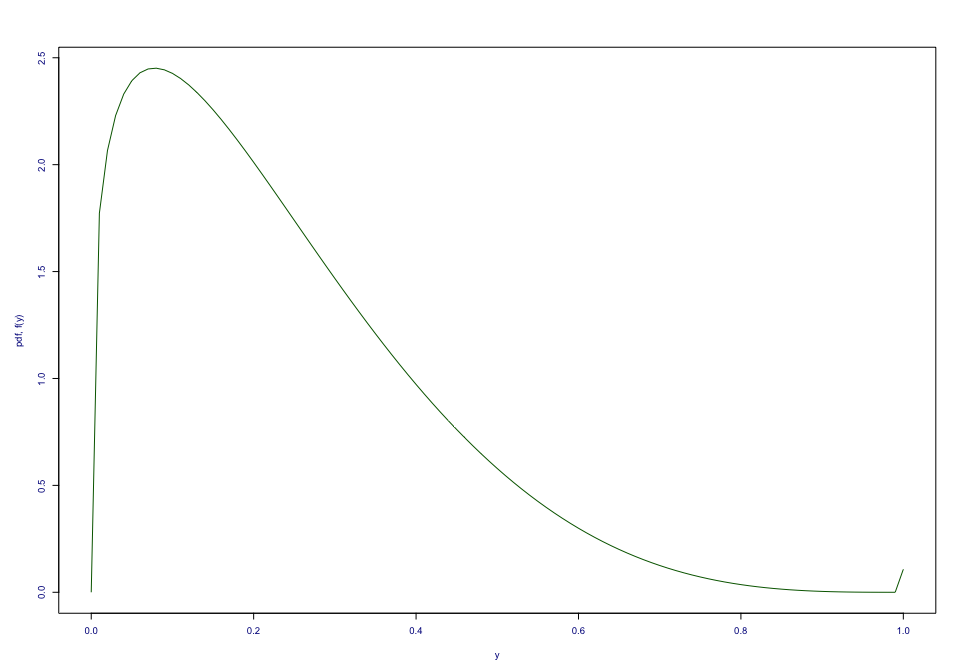


**Figure S12.** Mixture model for offer distribution in customer condition using expectation maximization to determine 2 constitutive distributions (top-bottom: normal: mu = 0.512, sigma = 0.0342; inflated beta: mu = 0.233, sigma = 0.394, nu = $6.67\times{10}^{-8}$, tau = 0.121).

## References

1. Tjur T. Coefficients of determination in logistic regression models – a new proposal: The coefficient of discrimination. The American Statistician [Internet]. 2009 Nov [cited 2017 Oct 5];63(4):366–72. Available from: <http://www.tandfonline.com/doi/abs/10.1198/tast.2009.08210>
